# Persistent Bacterial Coinfection of a COVID-19 Patient Caused by a Genetically Adapted *Pseudomonas aeruginosa* Chronic Colonizer

**DOI:** 10.3389/fcimb.2021.641920

**Published:** 2021-03-17

**Authors:** Jiuxin Qu, Zhao Cai, Yumei Liu, Xiangke Duan, Shuhong Han, Jihong Liu, Yuao Zhu, Zhaofang Jiang, Yingdan Zhang, Chao Zhuo, Yang Liu, Yingxia Liu, Lei Liu, Liang Yang

**Affiliations:** ^1^ Department of Clinical Laboratory, Shenzhen Third People's Hospital, Second Hospital Affiliated to Southern University of Science and Technology, National Clinical Research Center for Infectious Diseases, Shenzhen, China; ^2^ School of Medicine, Southern University of Science and Technology, Shenzhen, China; ^3^ Medical Research Center, Southern University of Science and Technology Hospital, Shenzhen, China; ^4^ The State Key Laboratory of Respiratory Diseases, The First Affiliated Hospital of Guangzhou Medical University, Guangzhou, China; ^5^ Shenzhen Key Laboratory of Pathogen and Immunity, State Key Discipline of Infectious Disease, Shenzhen Third People’s Hospital, Second Hospital Affiliated to Southern University of Science and Technology, Shenzhen, China; ^6^ Shenzhen Key Laboratory of Gene Regulation and Systems Biology, Southern University of Science and Technology, Shenzhen, China

**Keywords:** *Pseudomonas aeruginosa*, COVID-19, biofilm, coinfection, DNA methylation

## Abstract

*Pseudomonas aeruginosa* is a biofilm-forming opportunistic pathogen which causes chronic infections in immunocompromised patients and leads to high mortality rate. It is identified as a common coinfecting pathogen in COVID-19 patients causing exacerbation of illness. In our hospital, *P. aeruginosa* is one of the top coinfecting bacteria identified among COVID-19 patients. We collected a strong biofilm-forming *P. aeruginosa* strain displaying small colony variant morphology from a severe COVID-19 patient. Genomic and transcriptomic sequencing analyses were performed with phenotypic validation to investigate its adaptation in SARS-CoV-2 infected environment. Genomic characterization predicted specific genomic islands highly associated with virulence, transcriptional regulation, and DNA restriction-modification systems. Epigenetic analysis revealed a specific N_6_-methyl adenine (m_6_A) methylating pattern including methylation of alginate, flagellar and quorum sensing associated genes. Differential gene expression analysis indicated that this isolate formed excessive biofilm by reducing flagellar formation (7.4 to 1,624.1 folds) and overproducing extracellular matrix components including CdrA (4.4 folds), alginate (5.2 to 29.1 folds) and Pel (4.8–5.5 folds). In summary, we demonstrated that *P. aeuginosa* clinical isolates with novel epigenetic markers could form excessive biofilm, which might enhance its antibiotic resistance and *in vivo* colonization in COVID-19 patients.

## Introduction

Coronavirus Disease 2019 (COVID-19) is a fatal lung infection caused by the novel coronavirus named Severe Acute Respiratory Syndrome Coronavirus 2 (SARS-CoV-2) which has influenced millions of people globally since its onset. Its pathogenicity, epidemiology and treatments have been extensively studied (Chen et al., 2020; Li et al., 2020; Shen et al., 2020). It causes severe tissue damage of lungs and multiple organs such as the heart, the liver, and the kidneys (Wang D. et al., 2020; Wang T. et al., 2020). Higher mortality rate was observed from COVID-19 patients with older age and underlying diseases (Liu et al., 2020; Wang T. et al., 2020; Zhou et al., 2020). Such viral lung infection weakens host immunity and alters the composition and functions of respiratory microbiota, predisposing hosts to bacterial coinfections (Hanada et al., 2018). Bacterial coinfection was one of the major causes of death in the influenza pandemics last century (Hers et al., 1958; Bisno et al., 1971; Brundage and Shanks, 2008; Gill et al., 2010; Cilloniz et al., 2012). About 30% of cases was found to have bacterial coinfection in the 2009 H1N1 influenza pandemic even with antibiotic treatment (Gill et al., 2010; Hanada et al., 2018). Bacteria coinfecting with SARS-CoV-2 have also been reported by several retrospective studies based on cases from different geographical regions (Hughes et al., 2020; Lansbury et al., 2020; Rawson et al., 2020; Vaughn et al., 2020; Zhu et al., 2020). The common coinfecting bacterial species include *Haemophilus influenzae*, *Staphylococcus aureus*, *Klebsiella pneuminiae*, *Mycoplasma pneumonia*, *Streptococcus pneumoniae*, and *Pseudomonas aeruginosa* (Lansbury et al., 2020; Zhu et al., 2020). According to Lansbury et.al, *P. aeruginosa* is the second most frequently detected pathogen in COVID-19 patients based on the data included in their study (Lansbury et al., 2020).

In our hospital, *P. aeruginosa* is one of the commonest coinfecting bacteria in COVID-19 patients. It is a biofilm-forming opportunistic pathogen causing life-threatening chronic infections in immunocompromised individuals with diseases like burn wounds, urinary tract infections, and respiratory infections (Gellatly and Hancock, 2013). Biofilm formation leads to higher antimicrobial resistance and lower virulence allowing chronic colonization of *P. aeruginosa* in the host (Gellatly and Hancock, 2013). Previous study showed that Respiratory Syncytial Virus (RSV) infection increases iron availability and promotes biofilm formation of coinfecting *P. aeruginosa* for prolonged colonization in the host (Hendricks et al., 2016). Fatal clinical case due to coinfection of *P. aeruginosa* and influenza virus has been also reported before (Su et al., 2019). *P. aeruginosa* adopts genetic modification in genes such as *rpoN*, *lasR, fleQ*, and *mucA* to reduce virulence, enhance biofilm formation, and increase antimicrobial resistance for long term colonization in CF patients (Smith et al., 2006; Marvig et al., 2015). Attenuation of its virulence by adaptive modification in *lasR* and *mpl* genes has also reported in *P. aeruginosa* isolated from ventilator-associated pneumonia patients (Wang et al., 2017). Although *P. aeruginosa* coinfection has been reported, its adaptive modification for prolonged colonization and invasion in COVID-19 patients still remains unclear.

In this study, we focused on a small colony variant (SCV) strain of *P. aeruginosa* isolated from sputum and bronchioalveolar lavage fluid (BALF) samples of a critical COVID-19 patient. Genomic and transcriptomic sequencing were performed using Illumina and PacBio sequencing analysis to investigate its genetic modification. Genome-wide DNA methylation analysis revealed that genes involved in its flagellar formation, exopolysaccharide (EPS) biosynthesis and quorum sensing systems were methylated. RNA sequencing analysis showed that expression of flagellar-related genes were significantly downregulated whereas EPS-related genes were overexpressed. More notably, expression of *las* and *rhl* quorum sensing genes were significantly elevated. This is opposite from previous studies that *P. aeruginosa* attenuates its quorum sensing systems in the biofilm for escaping from host immune clearance (Marvig et al., 2015; Wang et al., 2017). Phenotypic tests validated that the isolate expresses higher *las* and *rhl* quorum sensing systems and forms excessive biofilm by reduction of motility and overproduction of EPS. This study demonstrated that *P. aeruginosa* adopts complex genetic adaptations in the SARS-CoV-2 infected environment for higher antimicrobial resistance, persistent colonization and disease induction. Our findings contribute to the prognosis of disease development and treatment decision to manage *P. aeruginosa* coinfection in COVID-19 patients.

## Materials and Methods

### Strain Isolation

Two *Pseudomonas aeruginosa* small colony isolates were collected from sputum samples and bronchioalveolar lavage fluids (BALF) of a critically illed COVID-19 patient during routine clinical tests. Blood indices were recorded including counts of white blood cells (WBC), neutrophils (N), lymphocytes (L), and levels of interleukin-6 (IL-6), C-reactive protein (CRP), and Procalcitonin (PCT).

### Ethical Statement

This work includes neither any identifiable human data nor direct participation of the patient. Bacterial samples were taken from routine microbiological tests of clinical respiratory samples. Blood indices were taken from the results of routine clinical blood examinations. This work is approved by the Ethics Committee of Shenzhen Third People’s Hospital, Second Hospital Affiliated to Southern University of Science and Technology [2020-184] and the Ethics Committee of Southern University of Science and Technology [20200069].

### Bacterial Strains and Growth Media

The isolates and all used *P. aeruginosa* strains and plasmids are listed in **Table 1**. Cultures were grown in LB broth or ABTGC medium supplemented with 10% TSB unless stated. ABTGC medium contains 0.2% glucose, 0.2% casamino acids, 0.1% MgCl_2_, 0.1% CaCl_2_, 0.1% FeCl_3_, and 10% A10 medium consisting of 15.1mM (NH_4_)_2_SO_4_, 33.7mM Na_2_HPO_4_•2H_2_O, 22 mM KH_2_PO_4_, and 0.05mM NaCl. Three hundred μg/ml carbenicillin was used for selecting strains carrying pUCP22-*cdrA::gfp* plasmid. Colony morphology of the isolates was imaged by Olympus (Mshot, China) with 8X magnification.

**Table 1:** Bacteria strains and plasmid used in this study.

Strains or plasmids	Relevant genotype and/or characteristics	Reference
***Pseudomonas aeruginosa *PAO1**	*Pseudomonas aeruginosa *ATCC	(Hentzer et al., 2002)
***Pseudomonas aeruginosa *LYSZa7**	SCV Isolate 1	This study
***Pseudomonas aeruginosa *LYSZa8**	SCV Isolate 2	This study
**PAO1Δ*lasI*Δ*rhlI***	PAO1 *lasI* and *rhlI* double mutant	(Hentzer et al., 2003)
**PAO1Δ*lasI*Δ*rhlI*/*P_lasB::gfp_***	PAO1 *lasI* and *rhlI* mutant containing *lasB-gfp*(ASV) reporter fusion	(Hansen et al., 2012)
**PAO1Δ*lasI*Δ*rhlI*/*P_rhlA::gfp_***	PAO1 *lasI* and *rhlI* mutant containing *rhlA-gfp*(ASV) reporter fusion	(Hansen et al., 2012)
**PAO1/*P_cdrA::gfp_***	PAO1 containing *cdrA-gfp *reporter fusion plasmid	This study
**LYSZa7/*P_cdrA::gfp_***	Isolate 1 containing *cdrA-gfp *reporter fusion* *plasmid	This study
**LYSZa8/*P_cdrA::gfp_***	Isolate 2 containing *cdrA-gfp *reporter fusion plasmid	This study
**pUCP22-*cdrA::gfp***	Plasmid carrying *cdrA::gfp*	(Rybtke et al., 2012)

### Motility Assays

Swimming motility of the isolates and *P. aeruginosa* PAO1 was tested on 0.3% LB agar plates. Swarming motility of the strains was tested on 0.5% agar plates supplemented with 0.8% nutrient broth and 0.5% glucose. Overnight cultures were diluted to OD_600nm_ of 0.01 and inoculated into the agar using sterile toothpicks for swimming while 2 µl of the inoculums were spotted onto the agar for swarming. The plates were incubated at 37°C statically for 14–16 h. Bacterial motility was imaged using Chemiluminescent (ChampChemiTM580, Sage, China).

### Biofilm Formation Assay

Overnight cultures of the isolates and *P. aeruginosa* PAO1 were diluted to OD_600nm_ 0.01 in fresh LB broth as innoculums. One hundred µl of the innoculums were aliquoted into 96-well microtiter plate in triplicates and incubated statically for 24 h at 37°C for biofilm formation. Biofilm was washed twice with ddH_2_O. One hundred twenty-five µl of 0.1% crystal violet (CV) was added to each well and incubated for 15 min at room temperature for staining. The wells were washed twice thoroughly with ddH_2_O and air-dried. CV stain was dissolved into 125 µl of 30% acetic acid. Relative biofilm biomass was quantified by measuring optical density of CV staining on a Tecan infinity pro200 microplate reader at 550 nm.

### Quorum Sensing Inhibition Assay

Overnight cultures of the isolates, *P. aeruginosa* PAO1, PAO1*ΔlasIΔrhlI*, and quorum sensing (QS) reporter strains were diluted to OD_600nm_ 0.01 in ABTGC medium with 10% TSB and cultured to OD_600nm_ 0.8~1.0. Supernatants of the isolates, *P. aeruginosa* PAO1, and PAO1*ΔlasIΔrhlI* were collected by centrifugation at 12,000 g for 2 min and sterilized by filtration. Fifty µl of the supernatants were added to 50 µl of cultures of QS reporter strains respectively in triplicates. Green fluorescence and OD_600nm_ were recorded for 16 h in Tecan infinity pro200 microplate reader for QS expression. The relative expression of *las* and *rhl* QS was quantified by GFP/OD.

### Construction of Cyclic-Di-GMP Reporter Strains

*P. aeruginosa* PAO1 and the isolates were transformed with plasmid pUCP22-*cdrA::gfp* by electroporation. The transformants were selected on LB agar plates containing 300 µg/ml carbenicillin. The plasmid in the transformants was extracted and run on electrophoresis gel for confirmation.

### Cyclic-Di-GMP Expression Assay

Overnight cultures of the cyclic-di-GMP reporter strains were diluted to OD_600nm_ 0.01 in ABTGC medium with 10% TSB as inoculums. One hundred μl of inoculums were loaded into 96-well microplate in triplicates and allowed to grow to stationary phase with green fluorescence and OD_600nm_ measured using a Tecan Infinite Pro2000 microplate reader. Relative cyclic-di-GMP levels expressed in the isolates and PAO1 was quantified by GFP/OD.

### Genomic Extraction and Sequencing

The isolates were cultured to early stationary phase. Genomic DNA of the isolates was extracted by AxyPerp Bacterial Genomic DNA Miniprep Kit (Corning, New York, USA) and Mabio Bacterial DNA Extraction Mini Kits (Mabio), respectively, using manufacturer’s protocol. For short-read sequencing, PCR-free libraries of extracted genomic DNA were prepared by VAHTSTM PCR-Free DNA Library Prep Kit for Illumina^®^ (Vazyme, China) following manufacturer’s protocol. Purified fragments were tagged with VAHTSTM DNA Adapters for Illumina^®^ (Vazyme, China). Quality of the libraries were tested using qPCR and Agilent Technologies 2100 Bioanalyzer. Genomic sequencing was performed on Illumina HiSeq X platform for paired end reads of 150 bp. For long read sequencing, DNA was fragmented with G-tubes (Covaris) and end-repaired to prepare SMRTbell DNA template libraries according to the manufacturer’s specification (PacBio, Menlo Park, USA). Genomic sequencing was performed on the Pacific Biosciences RSII sequencer (PacBio, Menlo Park, USA) according to standard protocols.

### Transcriptomic Extraction and Sequencing

The isolates and *P. aeruginosa* PAO1 were cultured to early stationary phase. Total RNA was extracted using Magen HiPure Universal RNA Mini kits (MCBio, China) according to the manufacturer’s instructions. Concentrations of RNA were measured by Qubit 2.0 (Thermo Fisher Scientific, MA, USA) and Nanodrop One (Thermo Fisher Scientific, MA, USA). RNA integrity was evaluated using Agilent 2100 system (Agilent Technologies, Waldbron, Germany). RNA libraries were prepared by NEB Next^®^ Ultra™ Directional RNA Library Prep Kit for Illumina^®^ (New England Biolabs, MA, USA) following manufacturer’s instruction. Ribosomal RNA were removed by Ribo-zero rRNA Removal Kit. cDNA was synthesized using NEB Next First Strand Synthesis Reaction Buffer. RNA sequencing was performed on Illumina NovaSeq 6000 platform for paired end reads of 150 bp.

### Sequencing Data Analysis

For genomic sequencing reads, Illumina reads were trimmed with automatic adaptor trimming option and assembled into contigs using De Novo Assembly module of CLC Genomics Workbench 20 (Qiagen) with default parameters. Phylogenetic tree was drawn by libMUSCLE aligment mode of Parsnp package using complete genomes (Treangen et al., 2014). PacBio reads were assembled into complete genome using HGAP4 pipeline of SMRTLink software v9.0 with default settings. Multilocus sequence typing (MLST) and identification of antimicrobial resistance genes (85% identity and 60% minimal length) using LYSZa7 genome were performed on the Center of Genomic of Epidermiology webserver (Larsen et al., 2012; Zankari et al., 2012). Circular plot was drawn using BLAST Ring Image Generator (Alikhan et al., 2011). Genomic islands on LYSZa7 genome were predicted by IslandViewer 4 webserver (Bertelli et al., 2017). LYSZa7 genome was annotated by prokka v1.14.6 (-species Pseudomonas aeruginosa –metagenome –Kingdom bacterium) (Seemann, 2014). DNA methylation analysis was performed using Base Modification Analysis and Motif Analysis application of SMRTLink software v9.0. The complete genome sequence was uploaded into the SMRT portal as reference sequence. A default modification quality value (QV) score of 30 (correspond to a p-value of 0.001) was used to call the modified bases. Restriction modification system genes were predicted and assigned to identified recognition motifs, with REBASE (Roberts et al., 2010). Grep function in shell was used to match methylated genes and QS and biofilm related genes.

Illumina RNA sequencing reads of the isolates were preprocessed and analyzed using RNA analysis module of CLC Genomics Workbench 20 (Qiagen) with default settings. Differential gene expression was done using Empirical Analysis of DGE module using selection criteria of absolute fold change ≧4 and adjusted p-value <0.05, with *P. aeruginosa* PAO1 as reference. GO enrichment analysis of significantly regulated genes was performed on DAVID bioinformatics database v6.8 (Ma et al., 2012). PCoA plot and heatmap were drawn using vegan, ggplot2, and pheatmap packages in R 4.0.0 software.

### Data Availability

All Illumina sequencing data used in this study could be found under BioProject No. PRJNA656063 and assembled genome of LYSZa7 could be found under BioProject No. PRJNA656096 on NCBI.

## Results

### *Pseudomonas aeruginosa* Coinfection and Immune Responses

**Figure 1:**
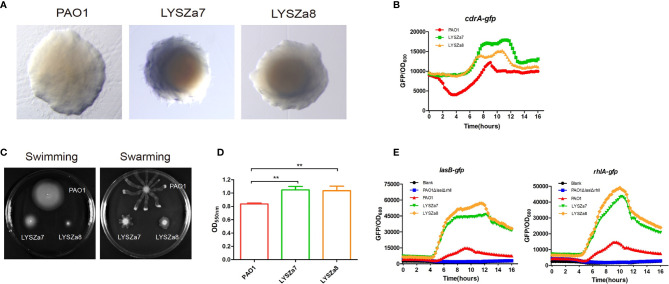
Phenotypic tests. **(A)** Colony morphologies of *P. aeruginosa* PAO1, LYSZa7 and LYSZa8; **(B)** Cyclic-di-GMP levels in *P. aeruginosa* PAO1, LYSZa7 and LYSZa8 measured by *cdrA*::gfp reporter assay; **(C)** Swimming motility (left plate) and swarming motility(right plate) of *P. aeruginosa* PAO1, LYSZa7 and LYSZa8; **(D)** Biofilm formation of *P. aeruginosa* PAO1, LYSZa7, and LYSZa8 quantified by CV staining, **p-value <0.05; **(E)** *las* and *rhl* quorum sensing expression measured by PAO1Δ*lasI*Δ*rhlI*/*P_lasB::gfp_* and PAO1Δ*lasI*Δ*rhlI*/*P_rhlA::gfp_* reporter strains.

**Figure 2:**
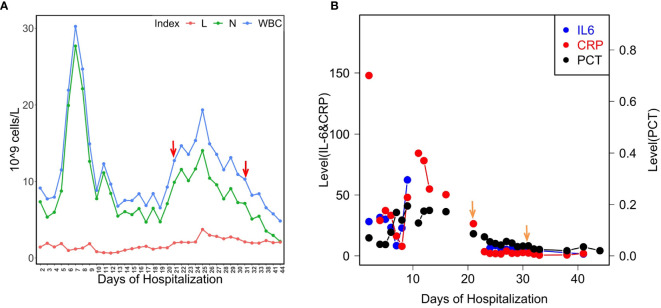
Levels of the patient’s blood indices. **(A)** Counts of WBC, L and N during the course of hospitalization. The isolates were found over the period of the second immune peak. Red arrows indicate the isolation times of LYSZa7(day 21) and LYSZa8(day 31)**; (B)** Levels of IL-6 (pg/ml), CRP (mg/L), and PCT (ng/ml) during the course of hospitalization. Orange arrows indicate the isolation times of LYSZa7(day 21) and LYSZa8(day 31).

In our hospital, *P. aeruginosa* is the third mostly identified coinfecting bacterium among COVID-19 patients. 5.1% (21/408) of all the COVID-19 patients was diagnosed with secondary infections, among which 23.8% (5/21, 4 critically illed and 1 severely illed patients) was infected with *P. aeruginosa* (unpublished data). Among all the *P. aeruginosa* isolates collected from these patients, two isolates collected from one critically illed patient exhibit small colony variant morphology with wrinkled edge and condensed extracellular matrix with stronger biofilm forming capacity (**Figures 1A, D**). We thus focused on these two isolates for further analysis. These two *P. aeruginosa* isolates were collected from routine clinical respiratory samples longitudinally with 10 days interval. The first isolate collected from sputum sample was named as LYSZa7 while the second was from BALF sample and named as LYSZa8. Counts of white blood cells (WBC), neutrophils (N), lymphocytes (L), and levels of interleukin-6 (IL-6), C-reactive protein (CRP) and Procalcitonin (PCT) were monitored as immune response indicators (**Figure 2**). Two peaks were observed from levels of WBC, N and L whereas the isolates were collected over the period of the second peak (**Figure 2A**). CRP is an effective diagnostic marker to differentiate viral and mixed/bacterial infection in respiratory infections. Significantly higher levels of CRP (> 40 mg/L) could be detected from patients with bacterial and influenza viral coinfection comparing to that of patients with sole influenza viral infection (Ahn et al., 2011; Li et al., 2019). High levels of CRP observed from day 2 to day 23 suggested that there should be viral and bacterial coinfection in the patient’s respiratory system (**Figure 2B**). The emergence of *P. aeruginosa* aligned well with the peak of immune response indicators. Similar trend was observed from the levels of immune response indicators of the other 3 critically illed patients (**Figure S1**). Thus, *P. aeruginosa* was highly associated with the bacterial coinfection induced in the patients during critical stage of COVID-19. We therefore further investigated the *P. aeruginosa* SCV isolates on their survival and adaptation in SARS-CoV-2 virus infected environment.

### Genomic and Epigenetic Characterization of *P. aeruginosa* SCV Isolates

Genomic sequencing was performed using Illumina and PacBio sequencing platforms to investigate the genomic characteristics of the isolates. MLST analysis using Illumina contigs showed that both isolates are of *P. aeruginosa* ST1445. LYSZa7 and LYSZa8 are of same sequencing type with different evolving times. Thus, we put our focus solely on LYSZa7 genome. Its complete genome is 6,534,364 bp in length with an average GC of 66.21%. It carries several antimicrobial resistance genes (ARGs) including *aph(3’)-IIb*, *bla_OXA-395_*, *bla_PAO_*, *fosA* and *catB7* against aminoglycoside, beta-lactam, fosfomycin, and phenicol drugs (**Figure 4**, outermost circle).

**Figure 3:**
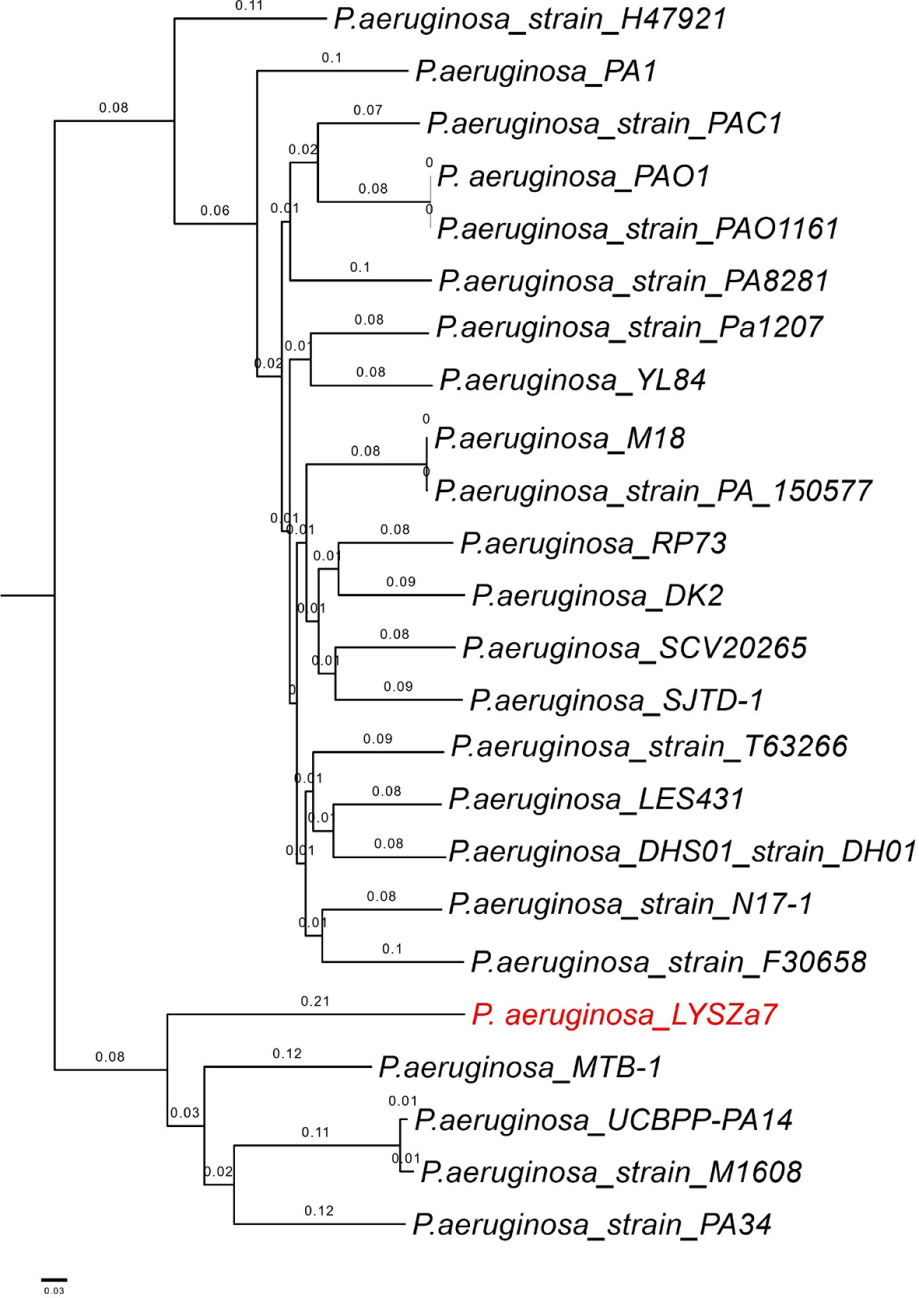
Phylogenetic tree constructed using whole genomes of LYSZa7 and 23 other *P. aeruginosa* laboratory, clinical and environmental strains selected from NCBI database. *P. aeruginosa* PAO1 was used as reference strain.

**Figure 4:**
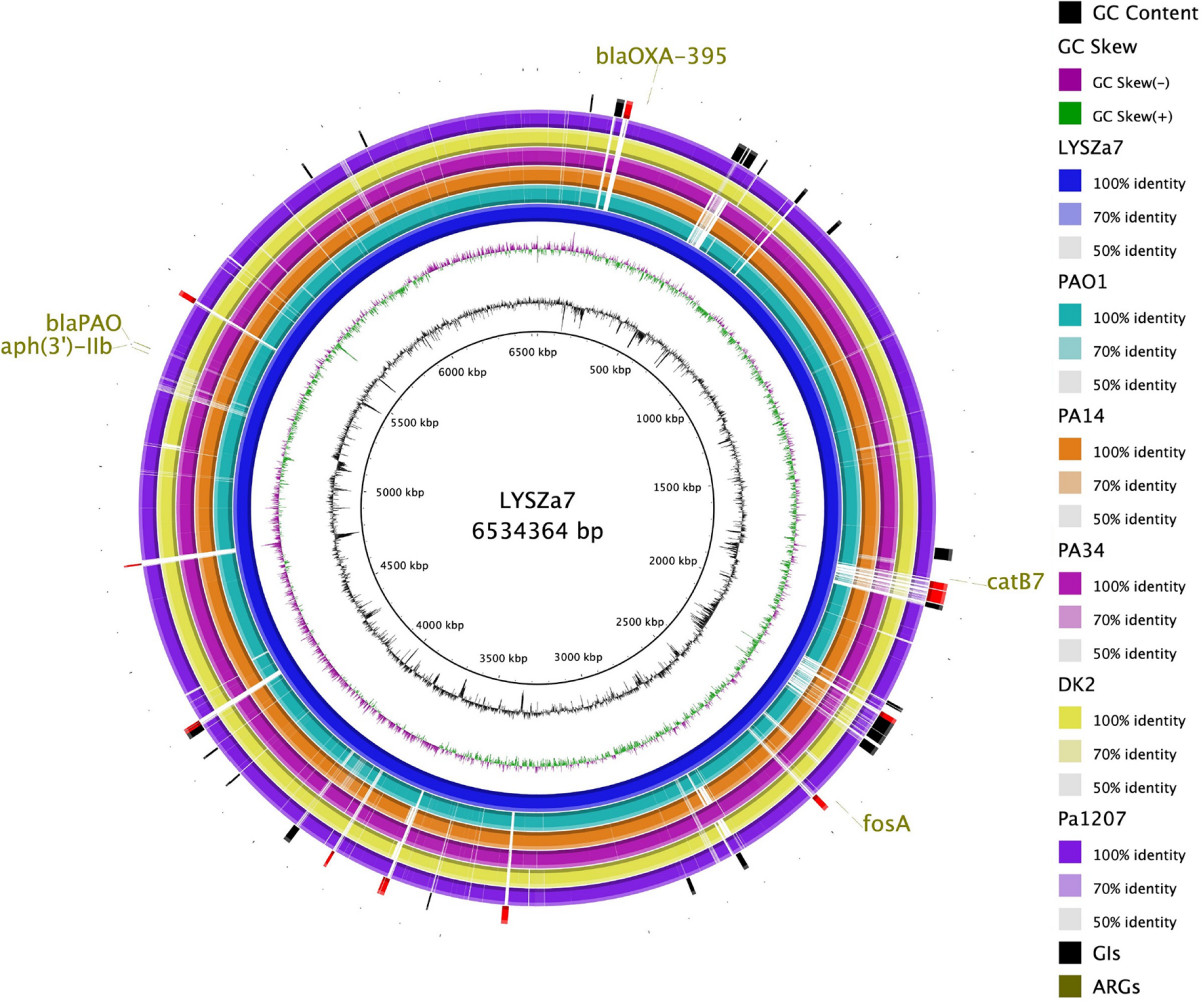
Circular plot. From the innermost, Circle 1: GC content; Circle 2: GC Skew; Circle 3: *P. aeruginosa* LYSZa7; Circle 4: *P. aeruginosa* PAO1; Circle 5: *P. aeruginosa* PA14 (virulent clinical isolate); Circle 6: *P. aeruginosa* PA34 (strain isolated from Keratitis patient); Circle 7: *P. aeruginosa* DK2 (Strain isolated from Cystic Fibrosis patient); Circle 8: *P. aeruginosa* Pa1207 (strain isolated from Bacteremia patient); Circle 9: Genomic Islands predicted with specific GIs of LYSZa7 highlighted in red; Circle 10: Antimicrobial resistance genes (ARGs).

Phylogenetic tree was constructed by comparing LYSZa7 with 23 other *P. aeruginosa* genomes selected from NCBI Genbank to trace its evolutionary origin (**Table S1**). LYSZa7 branched out individually with a closer distance to the virulent clinical strain PA14 while positioned further away from PAO1 reference strain and certain clinical strains such as DK2 isolated from cystic fibrosis patients (**Figure 3**). Based on the phylogenetic tree, genome of LYSZa7 was compared with selected species including PAO1 reference strain and virulent clinical strains, PA14, PA 34, DK2, and Pa1207 isolated from patients with different diseases like cystic fibrosis, keratitis, and bacteremia to predict specific genomic islands (GIs) on LYSZa7 genome (**Figure 4**). As seen from the circular plot, there are specific GIs present only on LYSZa7 genome (**Figure 4**, circle 9, highlighted in red). These GIs are involved in transposition, toxin transport, transcriptional regulation and especially DNA restriction-modification (R-M) system for survival, persistence, and invasion in the respiratory tract during coinfection (**Table S2**).

DNA methylation as part of DNA restriction-modification systems modifies the nucleotide without changing the genome sequence to regulate gene expression and control phenotypic traits in epigenetic perspective (Lobner-Olesen et al., 2005; Vasu and Nagaraja, 2013). Specific GIs for type I and type III DNA restriction-modification system were predicted on LYSZa7 genome. DNA methylation analysis indicated that genes related to *las* and *rhl* quorum sensing systems, alginate production and flagellar formation were methylated by two different methyltransferases from both R-M systems predicted (**Table 2**). DNA sequencing analysis revealed the phylogeny, unique GIs and a particular pattern of DNA methylation of LYSZa7. Transcriptomic analysis was then performed to evaluate gene expression of the isolates.

**Table 2:** Genes methylated in LYSZa7 comparing to *P. aeruginosa* PAO1 and motif information. Type: type of R-M system.

Type	Methyltransferase	Motif	Genes methylated*
Type III	M.PaeZa7II	CCCGAG	*algD algF algK fliC fliD lasB rhlB*
Type I	M.PaeZa7I	AGGNNNNNPTGT	*fliC*

*Grep function in shell was used to match methylated genes and QS and biofilm related genes.

### Pre-Transcriptional and Transcriptional Regulations Lead to Excessive Biofilm Formation and Elevated Quorum Sensing Systems

**Figure 5:**
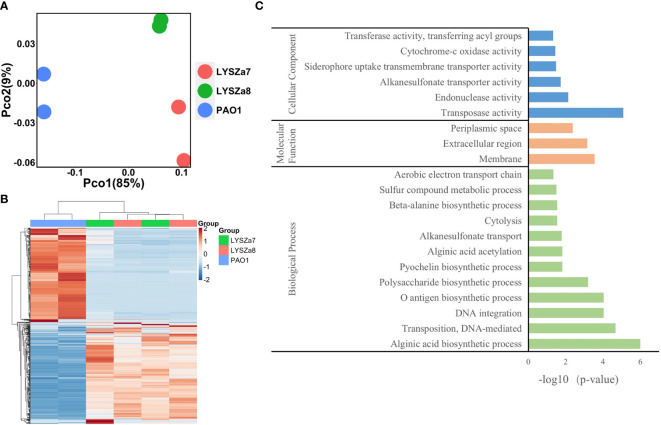
Dissimilarity analysis of samples and Gene Orthology analysis of differentially expressed genes; **(A)** PCoA plot of *P. aeruginosa* PAO1, LYSZa7 and LYSZa8 by Bray Curtis dissimilarity; **(B)** Heatmap of PAO1, LYSZa7 and LYSZa8 based on euclidean clustering distance; **(C)** Gene Orthology Enrichment based on the significantly regulated genes (fold change ≧ 4, adjusted p-value < 0.05) in LYSZa7 comparing to those of PAO1.

RNA sequencing was performed on Illumina sequencing platform and differential gene expression (DGE) in LYSZa7 and LYSZa8 was analyzed using *P. aeruginosa* PAO1 as reference to investigate transcriptional regulation during coinfection. We involved the second isolate here to learn the transcriptional versatility between two isolates. Clear separation between the isolates and PAO1 along PC1 (85%) on PCoA plot implied the significant differences in their transcriptomic profiles (**Figure 5A**). The proximity between clusters of the two isolates along both PC1 (85%) and PC2 (9%) implied the similarity in their gene expressions. Such differences among the transcriptomics profiles of PAO1 and the two isolates, and similarity between that of the two isolates could also be seen from heatmap plot (**Figure 5B**).

Results of DGE between LYSZa7 and LYSZa8 aligned with the dissimilarity analysis showing only minor variation in their gene expression profiles was observed. Only 39 genes were significantly downregulated in LYSZa8 comparing to LYSZa7 with mostly low mean counts (**Table S3**). None of the genes participate in biofilm formation and quorum sensing systems. This indicated that the isolates proliferated rather stably in the respiratory system during coinfection.

DGE of PAO1 was compared with that of LYSZa7 since there was only minimal difference between LYSZa7 and LYSZa8. In total, 481 genes were differentially regulated in LYSZa7 (**Table S4**). Among which, 38 genes were involved in biofilm formation and virulence regulation of the isolate (**Table 3**). Gene ontology analysis also demonstrated enrichment of pathways essential for biofilm formation and virulence such as polysaccharide/alginate biosynthesis and O antigen biosynthesis pathways (**Figure 5C**). In *P. aeruginosa*, the second messenger, cyclic-di-GMP promotes biofilm formation while it is controlled by two groups of enzymes, phosphodiesterase (PDE) for its degradation and diguanylate cyclase (DGC) for biosynthesis (Kulasakara et al., 2006). As *cdrA* is positively regulated by cyclic-di-GMP, overexpression of *cdrA* reflects an increase in cyclic-di-GMP biosynthesis (Borlee et al., 2010). In LYSZa7, expression of *cdrA* (PA4625) gene was upregulated for 4.41 folds indicating an accumulation of cyclic-di-GMP. *cdrA::gfp* reporter assay confirmed the excessive production of cyclic-di-GMP in both LYSZa7 and LYSZa8 (**Figure 1B**). Such cyclic-di-GMP accumulation is probably caused by the downregulation of the PDE gene, *arr*, which decreased for 318.93 folds. Cyclic-di-GMP in turn bound to the I-site on PA2771 inducing self-inhibition of this DGC and resulted in its downregulation (Hoffman et al., 2005; Chen et al., 2016) (**Table 3**). As cyclic-di-GMP promotes biofilm formation, we tested biofilm formation of LYSZa7 and LYSZa8 by CV staining assay. Results showed that LYSZa7 and LYSZa8 are indeed strong biofilm-formers as they formed significantly more biofilm comparing to the reference PAO1 strain (**Figure 1D**).

**Table 3:** Significantly regulated genes involved in virulence and biofilm formation in LYSZa7 comparing to *P. aeruginosa* PAO1 (fold change ≧ 2, FDR adjusted p-value < 0.05); means: normalized mean counts of the sample groups.

Feature ID	Product	Fold change	Adjusted p-value	PAO1 Means	LYSZa7 Means
*alg44*	alginate biosynthesis protein Alg44	11.17	3.14E-47	26.0	277.9
*alg8*	alginate biosynthesis protein Alg8	7.87	8.78E-57	53.3	389.8
*algA*	phosphomannose isomerase/guanosine 5’-diphospho-D-mannose pyrophosphorylase	29.12	1.16E-75	47.9	1352.3
*algD*	GDP-mannose 6-dehydrogenase AlgD	19.91	7.43E-60	56.3	1076.1
*algE*	Alginate production outer membrane protein AlgE precursor	15.83	8.60E-64	25.1	393.2
*algF*	alginate o-acetyltransferase AlgF	19.26	5.84E-56	14.5	322.3
*algI*	alginate o-acetyltransferase AlgI	5.19	2.60E-23	47.9	226.6
*algJ*	alginate o-acetyltransferase AlgJ	17.14	2.28E-46	4.8	182.5
*algK*	alginate biosynthetic protein AlgK precursor	14.33	8.76E-48	16.2	250.3
*algL*	poly(beta-d-mannuronate) lyase precursor AlgL	14.69	1.29E-58	21.6	321.1
*algX*	alginate biosynthesis protein AlgX	20.54	1.60E-59	10.8	308.2
*arr*	aminoglycoside response regulator	-318.93	1.25E-49	229.6	2.5
*flgL*	flagellar hook-associated protein type 3 FlgL	-8.39	1.14E-72	1,319.7	173.3
*fliC*	flagellin type B	-7.44	0.00E+00	33,619.2	6865.2
*fliD*	flagellar capping protein FliD	-1,624.06	8.80E-207	6,412.3	5.7
PA2771	diguanylate cyclase with a self-blocked I-site, Dcsbis	-17.32	2.07E-06	348.4	20.4
PA4625	cyclic diguanylate-regulated TPS partner A, CdrA	4.41	4.60E-17	971.8	5208.9
*pelD*	PelD	5.55	5.54E-35	104.8	561.9
*pelE*	PelE	4.80	6.62E-20	65.3	280.4
*pelF*	PelF	5.00	8.20E-27	88.6	414.8
*pilA*	type 4 fimbrial precursor PilA	-961.48	1.61E-221	29,476.2	40.7
*waaL*	O-antigen ligase, WaaL	-8.00	3.94E-10	545.0	71.8
*wbpA*	UDP-N-acetyl-d-glucosamine 6-Dehydrogenase	-1,275.14	1.09E-199	6,270.7	7.2
*wbpB*	UDP-2-acetamido-2-deoxy-d-glucuronic acid 3-dehydrogenase, WbpB	-1,832.88	2.71E-164	3,815.0	4.8
*wbpD*	UDP-2-acetamido-3-amino-2,3-dideoxy-d-glucuronic acid N-acetyltransferase, WbpD	-2,685.85	6.53E-163	1,683.3	3.4
*wbpE*	UDP-2-acetamido-2-dideoxy-d-ribo-hex-3-uluronic acid transaminase, wbpE	-2,156.03	6.33E-173	4,611.0	4.8
*wbpG*	LPS biosynthesis protein WbpG	-2,523.51	3.59E-141	4,083.4	4.2
*wbpH*	probable glycosyltransferase WbpH	-1,978.78	4.72E-166	2,238.8	3.7
*wbpI*	UDP-N-acetylglucosamine 2-epimerase WbpI	-2,058.27	6.90E-147	3,268.5	4.3
*wbpJ*	probable glycosyl transferase WbpJ	-1,336.39	9.86E-162	1,523.7	4.3
*wbpK*	probable NAD-dependent epimerase/dehydratase WbpK	-1,800.55	2.03E-149	1,150.5	3.7
*wbpL*	glycosyltransferase WbpL	-1,912.92	3.51E-164	1,218.6	3.8
*wzx*	O-antigen translocase	-638.58	3.01E-98	435.9	3.4
*wzy*	B-band O-antigen polymerase	-6,615.39	2.54E-138	757.6	3.2
*wzz*	O-antigen chain length regulator	-655.23	7.50E-152	1,113.7	4.5
*lasB*	elastase LasB	2.60	7.02E-06	4,484.2	15730.4
*rhlA*	rhamnosyltransferase chain A	2.74	1.41E-14	592.2	1887.9
*rhlB*	rhamnosyltransferase chain B	3.05	5.51E-16	294.8	940.1

Cyclic-di-GMP promotes biofilm formation through various mechanisms including reducing motility and enhancing biosynthesis of exopolysaccharides. Decrease in expression of genes involved in flagellar formation, *fliCD* and *flgL*, impaired swimming and swarming motility of the isolates (**Table 3**, **Figure 1C**). Moreover, expression of *al*g and *pel* genes for exopolysaccharide biosynthesis raised significantly for 4.8 to 29.12 folds (**Table 3**). Methylation of *fliCD* genes and *algDFK* genes indicated that these genes were regulated at pre-transcriptional level as well (**Table 2**). Reduction in motility and overproduction of exopolysaccharides, alginate and Pel, are major factors leading to excessive biofilm formation by the isolates for prolong colonization.

*P. aeruginosa* possesses several virulence factors for invasion and disease induction in hosts, such as quorum sensing systems, siderophore production and lipopolysaccharide (LPS) (Hentzer et al., 2003). Transcription of genes responsible for LPS biosynthesis (*waaL, wbp, wzx/y/z*) was greatly inhibited in LYSZa7 impairing LPS biosynthesis (**Table 3**). More notably, expression of quorum sensing genes, *lasB* and *rhlAB*, increased for 2.6, 2.74, and 3.05 folds, respectively, in LYSZa7 (**Table 2**). We thus collected the supernatants of the reference PAO1 strain, the isolates and quorum sensing mutant to test the expression of *lasB* and *rhlA* genes using *lasB::gfp* and *rhlA::gfp* reporter assays. Results clearly showed that the expression of *lasB* and *rhlA* genes was much higher in the isolates comparing to PAO1 and the mutant (**Figure 1E**). This is opposite from previous studies reporting that *P. aeruginosa* attenuated quorum sensing systems during colonization in the respiratory systems (Smith et al., 2006; Wang et al., 2017). Methylation of *lasB* and *rhlB* genes indicated that regulation of quorum sensing systems started pre-transcriptionally. Our results suggested that *P. aeruginosa* isolate remodels its biofilm forming capacity and dynamically adjusts its virulence to adapt to the SARS-CoV-2 infected environment in COVID-19 patients.

## Discussion

In this study, we characterized two *P. aeruginosa* SCVs isolated from respiratory samples of one critical COVID-19 patient on genomic, transcriptomic, and phenotypic levels for their adaptation and underlying mechanisms causing bacterial coinfection. The two isolates are of same sequence typing with relatively stable transcriptomic profiles. Characterization of LYSZa7 genome indicated that the isolates carry specific GIs for R-M systems and may result in DNA methylation. N_6_-methyl adenine (m_6_A) methylation was observed on genes involved in flagellar formation, alginate biosynthesis and quorum sensing systems based on the results of epigenetic analysis. RNA-seq analysis indicated that expression of 38 genes for biofilm formation and virulence were differentially regulated. Overlapped genes between epigenetic analysis and transcriptomic analysis showed that gene regulation may occur at both pre-transcriptional and transcriptional levels. We demonstrated here that the isolates reduced motility and increased exopolysaccharides biosynthesis to enhance biofilm formation, and reinforced *las* and *rhl* quorum sensing systems to colonize and persist in the COVID-19 patient.

Bacterial coinfection is complex process which has been frequently observed from patients with respiratory viral infections such as influenza pneumonia and resulted in high mortality rate. Viral infection damages tissues along respiratory track and regulates immune cells/cytokines resulting in dysbiosis of microbiome and facilitate bacterial colonization (McCullers, 2014; Bakaletz, 2017). In this study, fluctuated levels of immune cells (WBC, N, L) and blood indices (CRP and PCT) were recorded. CRP has been reported as an indicator of bacterial coinfection in viral pneumonia patients with a cut off value of more than 40 mg/L for mixed coinfection and lower for sole viral infection (Korppi and Kroger, 1993; Haran et al., 2013). With this as a reference, there should be viral and bacterial coinfection in the patient as the peak value of CRP reached more than 80 mg/L. Although there was no direct evidence to prove *P. aeruginosa* to be the causative pathogen, the emerging time of the isolates inferred a high correlation of this bacterium with bacterial coinfection in the patients based on the clinical data collected from all critical patients infected by *P. aeruginosa* (**Figures 2**, **S1**).

Previous studies revealed that viral infections facilitate bacterial colonization and promote biofilm formation. Influenza virus activates TGF-β and increase synthesis of cellular adhesins for enhancing attachment of group A *Streptococcus* to host cells (Li et al., 2015). RSV infection in the airway of CF patient activates antiviral interferon signaling which leads to higher iron availability and enhances *P. aeruginosa* biofilm formation (Hendricks et al., 2016). We observed similar phenomenon in this COVID-19 case that *P. aeruginosa* survived as small colony variant with enhanced biofilm formation in SARS-CoV-2 infected environment, by attenuating flagellar formation and enhancing exopolysaccharide production. In addition, phenotypic tests of *P. aeruginosa* isolates from the other three critically illed patients indicated obvious increases in biofilm formation in the later isolates as well (data not shown), suggesting elevated biofilm formation as a general traits evolved during coinfection with SARS-CoV-2 virus. We here put our focus solely on LYSZa7 and LYSZa8 as they exhibited small colony variant morphology and showed the strongest biofilm forming capacity among all the *P. aeruginosa* isolates collected.

Moreover, as reported previously, coinfecting pathogens either form biofilm with compromised virulence to escape from immune clearance or dispersed from biofilm gaining higher virulence for disease induction. Secretion of virulent factors upon biofilm dispersion in the coinfecting bacteria, such as *Streptococcus pneumoniae* and *Staphylococcus aureus*, upon influenza virus infection enabled a transition from non-invasive colonization to secondary bacterial infection in the patients (Pettigrew et al., 2014; Reddinger et al., 2016). It is also well-known that *P. aeruginosa* attenuates its virulence in the biofilm to escape from host immune clearance for chronic colonization (Smith et al., 2006). A recent research demonstrated that damaged mucus prevents *P. aeruginosa* biofilm dispersion and enhances the expression of virulence pathways by regulating motility, quorum sensing systems, and production of siderophore and toxins (Wheeler et al., 2019). These suggest that the virulence of *P. aeruginosa* biofilm is dependent on pathological changes in host tissues and variation in environmental conditions in the niches. We, for the first time, reported here that *P. aeruginosa* could express higher virulence in SARS-CoV-2 infected respiratory system by upregulating *las* and *rhl* quorum sensing systems. SARS-CoV-2 virus causes tissue damages including diffuse alveolar damage and shedding of epithelial cells of alveolar (Martines et al., 2020). Such defects in tissues and weakened host immunity after viral infection probably give chance to *P. aeruginosa* to elevate its virulence.

Besides quorum sensing systems, lipopolysaccharide is also a key virulence factor of *P. aeruginosa* inducing inflammation in the host (Pier, 2007). It has been reported that alginate, Psl and Pel share the same precursors, mannose-1-phosphate, with LPS (Ma et al., 2012). The increase in the exopolysaccharide of the isolates was probably due to the biosynthetic flux towards alginate and Pel leading to a reduction in LPS biosynthesis. Thus, LPS is probably not a major contributor to virulence during coinfection. Moreover, results of *in vitro* antimicrobial susceptibility tests indicated that the isolates were susceptible to various antibiotics, but rather resistant to continuous clinical antibiotic treatments and persisted in patients’ respiratory systems. Such observation suggested that increased drug resistance is predominantly due to biofilm formation which shields *P. aeruginosa* from antimicrobial attack. Our results suggested that *P. aeruginosa* adopts unique adaptations in COVID-19 pneumoniae patients for survival and disease induction. Future studies on a larger scale are necessary to provide further support of such adaptation observed here.

This study demonstrated that *P. aeruginosa*, as a top coinfecting pathogen, could form more biofilm and elevate quorum sensing systems for prolonged colonization and bacterial coinfection induction during critical stage of COVID-19 pneumonia. Understanding these genetic adaptations of *P. aeruginosa* would greatly contribute to the prognosis of disease development and treatment scheme decision to manage secondary infections induced by *P. aeruginosa* in COVID-19 patients.

## Data Availability Statement

The datasets presented in this study can be found in online repositories. The names of the repository/repositories and accession number(s) can be found in the article/**Supplementary Material**.

## Author Contributions

JQ and ZC contributed to data analysis and manuscript preparation. YuL, XD, SH, JL, YuZ, ZJ, and YiZ contributed to data collection, experimental design, and operation. CZ, YaL, YiL, LL, and LY contributed to study design and manuscript verification. All authors contributed to the article and approved the submitted version.

## Funding

This work was supported by Guangdong Natural Science Foundation for Distinguished Young Scholar [2020B1515020003] and Start-up Grants [Y01416106 and Y01416206] from the Southern University of Science and Technology (SUSTech) to LY, Science and Technology Program of Shenzhen [JCYJ20190809144005609] and Guangdong Basic and Applied Basic Research Foundation [2020A1515010586] to JQ, the Guangzhou Municipal Science and Technology Bureau [Project No. 201607020044] to CZ, Grant from Bill & Melinda Gates Foundation to LL, Natural Science Fund of Guangdong Province [2020A1515010316] to YaL and Guangdong Basic and Applied Basic Research Foundation[2019A1515110640] to YiZ.

## Conflict of Interest

The authors declare that the research was conducted in the absence of any commercial or financial relationships that could be construed as a potential conflict of interest.

## Supplementary Material

Supplementary Figure 1 Blood index levels of the other 3 critically illed patients. **(A)** Blood index levels of patient 2: Upper panel: Counts of WBC, L and N during the course of hospitalization. Red arrows indicate the isolation times of first *P. aeruginosa* isolate (day 12) and last *P. aeruginosa* isolate (day 48); Lower panel: Levels of IL-6(pg/mL), CRP (mg/L) and PCT (ng/mL) during the course of hospitalization. Orange arrows indicate the isolation times of first *P. aeruginosa* isolate (day 12) and last *P. aeruginosa* isolate (day 48); **(B)** Blood index levels of patient 3: Upper panel: Counts of WBC, L and N during the course of hospitalization. Red arrows indicate the isolation times of first *P. aeruginosa* isolate (day 17) and last *P. aeruginosa* isolate (day 39); Lower panel: Levels of IL-6(pg/mL), CRP (mg/L) and PCT (ng/mL) during the course of hospitalization. Orange arrows indicate the isolation times of first *P. aeruginosa* isolate (day 17) and last *P. aeruginosa* isolate (day 39); **(C)** Blood index levels of patient 4: Upper panel: Counts of WBC, L and N during the course of hospitalization. Red arrows indicate the isolation times of first *P. aeruginosa* isolate (day 31) and last *P. aeruginosa* isolate (day 43); Lower panel: Levels of IL-6(pg/mL), CRP (mg/L) and PCT (ng/mL) during the course of hospitalization. Orange arrows indicate the isolation times of first *P. aeruginosa* isolate (day 31) and last *P. aeruginosa* isolate (day 43).
