# Supplementary figures and images for: Persistent Bacterial Coinfection of a COVID-19 Patient Caused by a Genetically Adapted Pseudomonas aeruginosa Chronic Colonizer
Source: Front Cell Infect Microbiol. 2021 Mar 17;11:641920. doi: 10.3389/fcimb.2021.641920 (PMC8010185; doi:10.3389/fcimb.2021.641920)

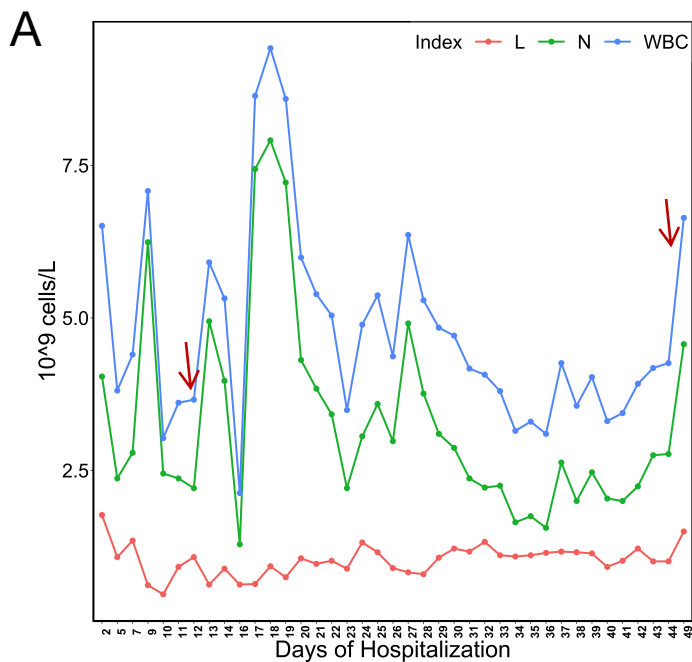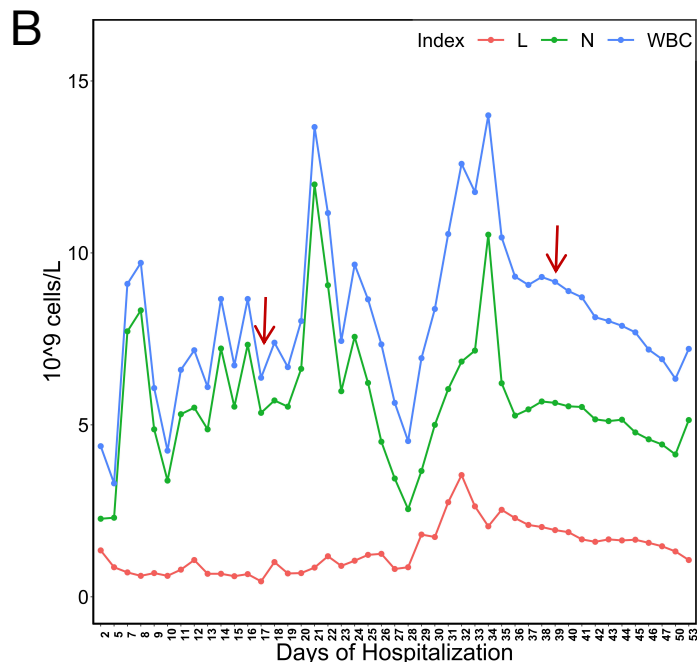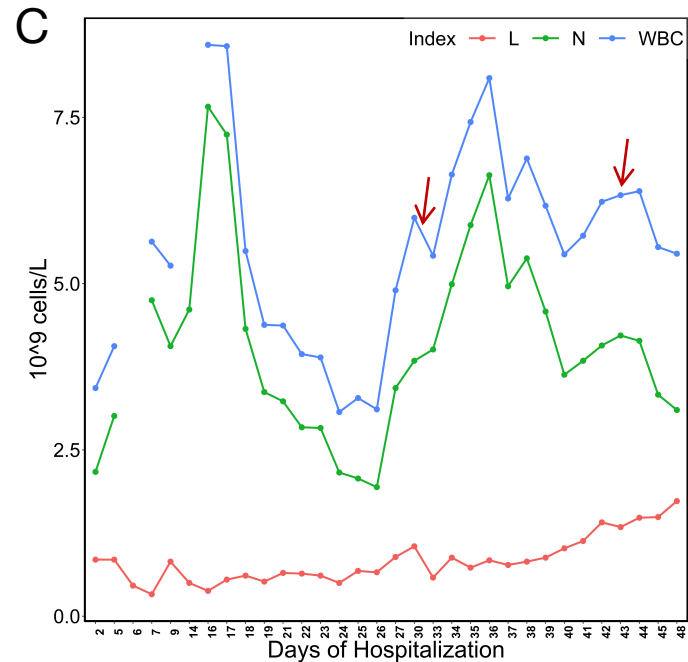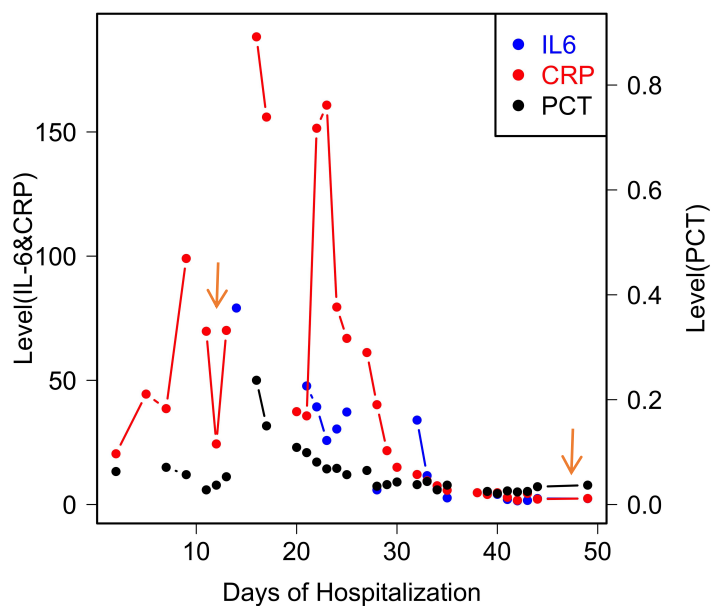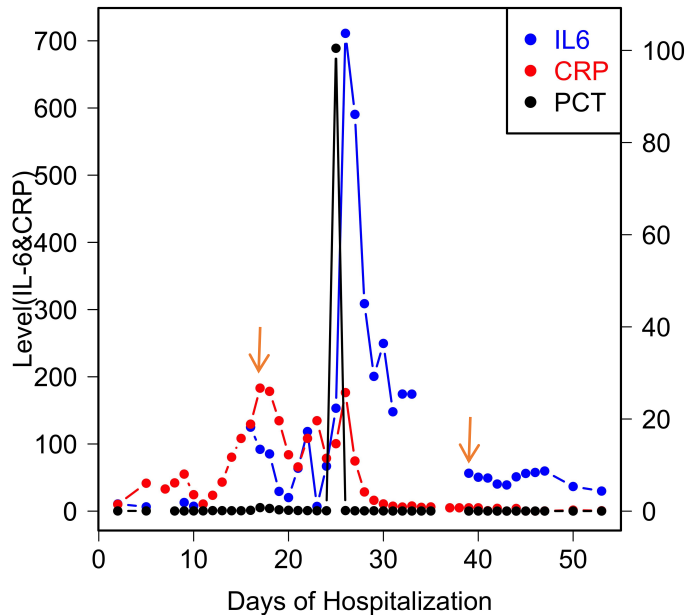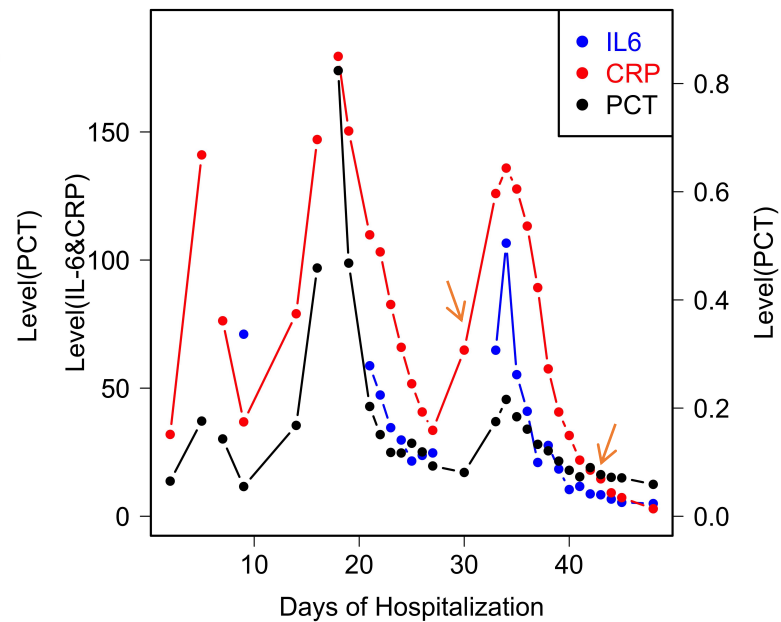

Supplement: Supplementary Figure 1 — Blood index levels of the other 3 critically illed patients. (A) Blood index levels of patient 2: Upper panel: Counts of WBC, L and N during the course of hospitalization. Red arrows indicate the isolation times of first P. aeruginosa isolate (day 12) and last P. aeruginosa isolate (day 48); Lower panel: Levels of IL-6(pg/mL), CRP (mg/L) and PCT (ng/mL) during the course of hospitalization. Orange arrows indicate the isolation times of first P. aeruginosa isolate (day 12) and last P. aeruginosa isolate (day 48); (B) Blood index levels of patient 3: Upper panel: Counts of WBC, L and N during the course of hospitalization. Red arrows indicate the isolation times of first P. aeruginosa isolate (day 17) and last P. aeruginosa isolate (day 39); Lower panel: Levels of IL-6(pg/mL), CRP (mg/L) and PCT (ng/mL) during the course of hospitalization. Orange arrows indicate the isolation times of first P. aeruginosa isolate (day 17) and last P. aeruginosa isolate (day 39); (C) Blood index levels of patient 4: Upper panel: Counts of WBC, L and N during the course of hospitalization. Red arrows indicate the isolation times of first P. aeruginosa isolate (day 31) and last P. aeruginosa isolate (day 43); Lower panel: Levels of IL-6(pg/mL), CRP (mg/L) and PCT (ng/mL) during the course of hospitalization. Orange arrows indicate the isolation times of first P. aeruginosa isolate (day 31) and last P. aeruginosa isolate (day 43). [file Image_1.pdf]
