# Supplementary material for: Persistent Bacterial Coinfection of a COVID-19 Patient Caused by a Genetically Adapted Pseudomonas aeruginosa Chronic Colonizer
Source: Front Cell Infect Microbiol. 2021 Mar 17;11:641920. doi: 10.3389/fcimb.2021.641920 (PMC8010185; doi:10.3389/fcimb.2021.641920)
Supplement: Supplementary file 5 [file Table_4.docx]

| **Feature ID** | **Product** | **Fold change** | **p-value** | **Adjusted p-value** | **PAO1 Means** | **LYSZa7 Means** |
| --- | --- | --- | --- | --- | --- | --- |
| *acoB* | acetoin catabolism protein AcoB | -6.99 | 6.17E-06 | 2.43E-05 | 48.3 | 6.0 |
| *acoR* | transcriptional regulator AcoR | -15.96 | 2.86E-06 | 1.19E-05 | 354.1 | 21.4 |
| *alg44* | alginate biosynthesis protein Alg44 | 11.17 | 1.15E-48 | 3.14E-47 | 26.0 | 277.9 |
| *alg8* | alginate biosynthesis protein Alg8 | 7.87 | 3.01E-58 | 8.78E-57 | 53.3 | 389.8 |
| *algA* | phosphomannose isomerase / guanosine 5'-diphospho-D-mannose pyrophosphorylase | 29.12 | 3.12E-77 | 1.16E-75 | 47.9 | 1352.3 |
| *algD* | GDP-mannose 6-dehydrogenase AlgD | 19.91 | 2.48E-61 | 7.43E-60 | 56.3 | 1076.1 |
| *algE* | Alginate production outer membrane protein AlgE precursor | 15.83 | 2.81E-65 | 8.60E-64 | 25.1 | 393.2 |
| *algF* | alginate o-acetyltransferase AlgF | 19.26 | 2.01E-57 | 5.84E-56 | 14.5 | 322.3 |
| *algI* | alginate o-acetyltransferase AlgI | 5.19 | 1.32E-24 | 2.60E-23 | 47.9 | 226.6 |
| *algJ* | alginate o-acetyltransferase AlgJ | 17.14 | 8.47E-48 | 2.28E-46 | 4.8 | 182.5 |
| *algK* | alginate biosynthetic protein AlgK precursor | 14.33 | 3.18E-49 | 8.76E-48 | 16.2 | 250.3 |
| *algL* | poly(beta-d-mannuronate) lyase precursor AlgL | 14.69 | 4.35E-60 | 1.29E-58 | 21.6 | 321.1 |
| *algX* | alginate biosynthesis protein AlgX | 20.54 | 5.37E-61 | 1.60E-59 | 10.8 | 308.2 |
| *armR* | antirepressor for MexR, ArmR | 4.97 | 2.42E-05 | 8.58E-05 | 1.7 | 20.4 |
| *arr* | aminoglycoside response regulator | -318.93 | 4.49E-51 | 1.25E-49 | 229.6 | 2.5 |
| *ccoN1* | Cytochrome c oxidase, cbb3-type, CcoN subunit | -4.29 | 0.00E+00 | 0.00E+00 | 6331.0 | 1958.4 |
| *cioA* | cyanide insensitive terminal oxidase | -6.04 | 3.42E-66 | 1.07E-64 | 1840.2 | 357.5 |
| *cioB* | cyanide insensitive terminal oxidase | -4.25 | 5.84E-25 | 1.17E-23 | 1442.4 | 398.0 |
| *clpV1* | ClpV1 | 6.33 | 3.94E-46 | 1.04E-44 | 1587.0 | 12659.8 |
| *coaB* | coat protein B of bacteriophage Pf1 | -113.14 | 4.46E-85 | 1.84E-83 | 457.1 | 3.7 |
| *czcC* | outer membrane protein precursor CzcC | 9.52 | 1.84E-22 | 3.45E-21 | 8.1 | 119.9 |
| *eraR* | response regulator EraR | 6.20 | 2.51E-07 | 1.24E-06 | 2.1 | 29.8 |
| *exaC* | NAD+ dependent aldehyde dehydrogenase ExaC | 111.19 | 4.44E-100 | 2.07E-98 | 21.4 | 2687.9 |
| *exoS* | exoenzyme S | -45.07 | 9.96E-63 | 3.00E-61 | 396.4 | 10.9 |
| *exoY* | adenylate cyclase ExoY | -16.56 | 1.54E-12 | 1.46E-11 | 56.8 | 2.7 |
| *fgtA* | flagellar glycosyl transferase, FgtA | -58.00 | 3.37E-134 | 1.88E-132 | 4377.4 | 84.2 |
| *fhp* | flavohemoprotein | 15.00 | 4.11E-86 | 1.72E-84 | 72.3 | 1031.3 |
| *fis* | DNA-binding protein Fis | -4.08 | 3.95E-22 | 7.33E-21 | 301.8 | 69.7 |
| *flgL* | flagellar hook-associated protein type 3 FlgL | -8.39 | 3.21E-74 | 1.14E-72 | 1319.7 | 173.3 |
| *fliC* | flagellin type B | -7.44 | 0.00E+00 | 0.00E+00 | 33619.2 | 6865.2 |
| *fliD* | flagellar capping protein FliD | -1624.06 | 1.10E-208 | 8.80E-207 | 6412.3 | 5.7 |
| *fpr* |  | -6.34 | 8.57E-76 | 3.11E-74 | 3336.3 | 645.1 |
| *fptA* | Fe(III)-pyochelin outer membrane receptor precursor | -13.76 | 7.94E-23 | 1.51E-21 | 1684.4 | 136.3 |
| *fpvA* | ferripyoverdine receptor | -30.09 | 2.55E-07 | 1.26E-06 | 2015.2 | 75.9 |
| *fusA1* | elongation factor G | -4.09 | 0.00E+00 | 0.00E+00 | 90369.2 | 34688.4 |
| *gcdH* | glutaryl-CoA dehydrogenase | 4.21 | 6.42E-05 | 2.10E-04 | 1476.6 | 7918.8 |
| *glpD* | glycerol-3-phosphate dehydrogenase | 4.83 | 1.09E-16 | 1.53E-15 | 427.7 | 2357.7 |
| *groEL* | GroEL protein | -4.01 | 1.91E-14 | 2.16E-13 | 70420.2 | 29017.8 |
| *hcp1* | Hcp1 | 7.35 | 3.84E-74 | 1.36E-72 | 2768.0 | 28878.3 |
| *hcpB* | secreted protein Hcp | -7.25 | 2.28E-43 | 5.87E-42 | 1690.4 | 268.8 |
| *hisF2* | imidazoleglycerol-phosphate synthase, cyclase subunit | -11220.44 | 1.18E-146 | 7.13E-145 | 1243.7 | 0.5 |
| *hisH2* | glutamine amidotransferase | -4086.10 | 1.18E-80 | 4.46E-79 | 476.0 | 0.5 |
| *hutU* | urocanase | 4.40 | 7.83E-21 | 1.35E-19 | 172.9 | 767.7 |
| *imm2* | pyocin S2 immunity protein | -1183.91 | 2.54E-90 | 1.10E-88 | 768.5 | 2.5 |
| *katN* | non-heme catalase KatN | -9.39 | 8.68E-07 | 3.89E-06 | 31.5 | 2.7 |
| *kdpB* | potassium-transporting ATPase, B chain | 7.68 | 6.01E-29 | 1.31E-27 | 61.8 | 439.3 |
| *kdpC* | potassium-transporting ATPase, C chain | 11.05 | 1.14E-13 | 1.20E-12 | 4.0 | 96.2 |
| *lecA* | LecA | 6.07 | 1.05E-14 | 1.21E-13 | 36.4 | 203.8 |
| *lpdV* | lipoamide dehydrogenase-Val | 4.24 | 1.60E-16 | 2.20E-15 | 4153.4 | 24849.6 |
| *mdcA* | malonate decarboxylase alpha subunit | 5.90 | 3.49E-46 | 9.29E-45 | 166.3 | 989.1 |
| *metF* | 5,10-methylenetetrahydrofolate reductase | -4.38 | 0.00E+00 | 0.00E+00 | 1847.8 | 502.7 |
| *metK* | methionine adenosyltransferase | -5.81 | 0.00E+00 | 0.00E+00 | 8099.2 | 1879.6 |
| *mexC* | Resistance-Nodulation-Cell Division (RND) multidrug efflux membrane fusion protein MexC precursor | 32.32 | 1.49E-07 | 7.65E-07 | 123.8 | 4017.2 |
| *mexD* | Resistance-Nodulation-Cell Division (RND) multidrug efflux transporter MexD | 26.91 | 3.47E-06 | 1.42E-05 | 283.8 | 9164.2 |
| *modA* | molybdate-binding periplasmic protein precursor ModA | 4.41 | 1.42E-18 | 2.25E-17 | 63.2 | 251.8 |
| *msuD* | methanesulfonate sulfonatase MsuD | 4.99 | 1.31E-07 | 6.75E-07 | 2.8 | 34.7 |
| *msuE* | NADH-dependent FMN reductase MsuE | 5.04 | 7.31E-04 | 1.97E-03 | 1.3 | 13.3 |
| *nadE* | NH3-dependent NAD synthetase | -4.57 | 0.00E+00 | 0.00E+00 | 3109.3 | 817.0 |
| *nirC* | probable c-type cytochrome precursor | 4.67 | 1.02E-20 | 1.74E-19 | 44.7 | 189.4 |
| *nirL* | heme d1 biosynthesis protein NirL | 4.65 | 5.66E-11 | 4.53E-10 | 26.6 | 123.8 |
| *norB* | nitric-oxide reductase subunit B | 23.22 | 5.16E-88 | 2.18E-86 | 86.8 | 2017.8 |
| *norC* | nitric-oxide reductase subunit C | 40.25 | 2.02E-102 | 9.52E-101 | 20.0 | 854.8 |
| *nosD* | NosD protein | 6.93 | 3.70E-42 | 9.46E-41 | 42.8 | 275.8 |
| *nosF* | NosF protein | 4.05 | 3.45E-09 | 2.24E-08 | 21.4 | 83.3 |
| *nosR* | regulatory protein NosR | 22.63 | 3.23E-96 | 1.45E-94 | 74.3 | 1617.5 |
| *nosZ* | nitrous-oxide reductase precursor | 13.20 | 1.14E-89 | 4.91E-88 | 68.2 | 846.3 |
| *ohr* | organic hydroperoxide resistance protein | -4.29 | 4.45E-17 | 6.42E-16 | 320.8 | 73.3 |
| *oprC* | Putative copper transport outer membrane porin OprC precursor | -7.25 | 0.00E+00 | 0.00E+00 | 4460.0 | 755.1 |
| *oprJ* | Multidrug efflux outer membrane protein OprJ precursor | 12.79 | 3.00E-05 | 1.05E-04 | 232.6 | 3137.2 |
| *osmE* | osmotically inducible lipoprotein OsmE | 4.09 | 2.72E-19 | 4.41E-18 | 495.8 | 2327.8 |
| *PA0048* | probable transcriptional regulator | 4.01 | 6.35E-21 | 1.11E-19 | 148.5 | 596.2 |
| *PA0049* | hypothetical protein | 4.23 | 5.96E-17 | 8.55E-16 | 600.0 | 2935.7 |
| *PA0053* | hypothetical protein | -11.99 | 4.31E-08 | 2.38E-07 | 360.4 | 30.8 |
| *PA0072* | TagS1 | 5.13 | 5.43E-40 | 1.36E-38 | 185.8 | 969.4 |
| *PA0073* | TagT1 | 4.21 | 6.67E-26 | 1.34E-24 | 213.1 | 917.8 |
| *PA0082* | TssA1 | 4.90 | 3.22E-33 | 7.68E-32 | 266.6 | 1382.6 |
| *PA0083* | TssB1 | 4.05 | 1.95E-15 | 2.41E-14 | 1008.7 | 4972.8 |
| *PA0084* | TssC1 | 4.35 | 1.03E-18 | 1.62E-17 | 2781.7 | 16930.2 |
| *PA0086* | TagJ1 | 8.36 | 8.72E-68 | 2.77E-66 | 444.9 | 4229.1 |
| *PA0087* | TssE1 | 8.74 | 9.32E-69 | 3.05E-67 | 114.7 | 985.9 |
| *PA0088* | TssF1 | 5.17 | 2.67E-34 | 6.48E-33 | 450.0 | 2639.2 |
| *PA0089* | TssG1 | 5.00 | 1.47E-32 | 3.47E-31 | 257.3 | 1346.3 |
| *PA0097* | hypothetical protein | 5.35 | 1.42E-31 | 3.24E-30 | 202.2 | 1102.7 |
| *PA0098* | hypothetical protein | -4.55 | 1.09E-11 | 9.43E-11 | 117.8 | 23.3 |
| *PA0099* | type VI effector protein | -5.19 | 6.47E-17 | 9.26E-16 | 235.1 | 40.7 |
| *PA0100* | hypothetical protein | -4197.29 | 7.13E-100 | 3.27E-98 | 489.7 | 0.5 |
| *PA0130* | 3-Oxopropanoate dehydrogenase | -7.45 | 0.00E+00 | 0.00E+00 | 4233.8 | 690.8 |
| *PA0131* | BauB | -6.18 | 3.97E-32 | 9.20E-31 | 489.3 | 81.9 |
| *PA0132* | Beta-alanine:pyruvate transaminase | -8.24 | 0.00E+00 | 0.00E+00 | 3940.8 | 587.8 |
| *PA0187* | hypothetical protein | -26.35 | 7.98E-22 | 1.45E-20 | 89.2 | 4.3 |
| *PA0188* | hypothetical protein | -11.02 | 2.28E-09 | 1.52E-08 | 45.3 | 3.2 |
| *PA0202* | probable amidase | -469.70 | 1.63E-59 | 4.82E-58 | 328.3 | 2.5 |
| *PA0203* | probable binding protein component of ABC transporter | -358.63 | 4.24E-16 | 5.63E-15 | 49.0 | 0.5 |
| *PA0204* | probable permease of ABC transporter | -261.87 | 7.24E-12 | 6.40E-11 | 34.8 | 0.5 |
| *PA0205* | probable permease of ABC transporter | -368.10 | 2.84E-15 | 3.49E-14 | 50.2 | 0.5 |
| *PA0206* | probable ATP-binding component of ABC transporter | -325.56 | 4.99E-16 | 6.59E-15 | 43.8 | 0.5 |
| *PA0207* | probable transcriptional regulator | -2499.05 | 1.04E-76 | 3.83E-75 | 307.9 | 0.5 |
| *PA0257* | hypothetical protein | -5.92 | 8.24E-03 | 1.74E-02 | 163.8 | 24.8 |
| *PA0258* | hypothetical protein | -16.83 | 4.74E-07 | 2.23E-06 | 22.0 | 1.8 |
| *PA0259* | Type 6 lipase adaptor, Tla3 | -20.11 | 7.46E-05 | 2.40E-04 | 930.9 | 48.7 |
| *PA0260* | Type 6 lipase effector, Tle3 | -34.11 | 3.30E-07 | 1.59E-06 | 1276.9 | 42.5 |
| *PA0261* | Type 6 lipase immunity, Tli3 | -19.07 | 3.33E-13 | 3.35E-12 | 179.1 | 8.8 |
| *PA0320* | calcium-regulated OB-fold protein CarO | 11.14 | 5.91E-81 | 2.25E-79 | 61.4 | 644.5 |
| *PA0327* | calcium-regulated beta-propeller protein CarP | 4.86 | 3.77E-32 | 8.77E-31 | 107.7 | 510.1 |
| *PA0399* | cystathionine beta-synthase | -4.08 | 0.00E+00 | 0.00E+00 | 3835.3 | 1174.8 |
| *PA0431* | hypothetical protein | -4.37 | 1.98E-32 | 4.67E-31 | 1619.2 | 438.4 |
| *PA0445* | probable transposase | 6.64 | 9.25E-06 | 3.53E-05 | 2.1 | 29.2 |
| *PA0457.1* | hypothetical membrane protein | -744.15 | 5.34E-27 | 1.11E-25 | 99.5 | 0.5 |
| *PA0493* | probable biotin-requiring enzyme | -4.12 | 5.90E-04 | 1.62E-03 | 143.4 | 32.4 |
| *PA0494* | probable acyl-CoA carboxylase subunit | -4.29 | 2.45E-06 | 1.03E-05 | 793.9 | 201.6 |
| *PA0497* | hypothetical protein | -4.33 | 4.05E-06 | 1.64E-05 | 153.2 | 32.2 |
| *PA0498* | hypothetical protein | -5.28 | 5.93E-08 | 3.22E-07 | 60.9 | 11.0 |
| *PA0505* | hypothetical protein | -4.24 | 7.55E-22 | 1.37E-20 | 423.1 | 101.3 |
| *PA0521* | probable cytochrome c oxidase subunit | 4.50 | 1.05E-12 | 1.02E-11 | 28.8 | 117.9 |
| *PA0522* | hypothetical protein | 6.22 | 1.72E-06 | 7.40E-06 | 1.7 | 22.7 |
| *PA0525* | probable dinitrification protein NorD | 5.96 | 4.58E-34 | 1.11E-32 | 279.9 | 1799.0 |
| *PA0529* | conserved hypothetical protein | -6.96 | 5.73E-77 | 2.12E-75 | 2164.8 | 369.2 |
| *PA0534* | FAD-dependent oxidoreductase | 11.40 | 4.02E-85 | 1.67E-83 | 232.1 | 2756.8 |
| *PA0535* | probable transcriptional regulator | 4.75 | 8.52E-45 | 2.23E-43 | 140.7 | 665.4 |
| *PA0561* | hypothetical protein | -11.63 | 4.37E-05 | 1.47E-04 | 325.0 | 28.0 |
| *PA0603* | AgtA | -4.97 | 2.43E-03 | 5.84E-03 | 2212.4 | 513.6 |
| *PA0604* | AgtB | -4.07 | 1.06E-06 | 4.70E-06 | 3924.7 | 1187.0 |
| *PA0633* | hypothetical protein | -20240.88 | 1.56E-170 | 1.09E-168 | 2180.6 | 0.5 |
| *PA0634* | hypothetical protein | -1660.15 | 4.37E-136 | 2.51E-134 | 1066.0 | 1.4 |
| *PA0635* | hypothetical protein | -5248.32 | 2.23E-117 | 1.11E-115 | 603.5 | 0.5 |
| *PA0636* | hypothetical protein | -25987.93 | 2.09E-171 | 1.49E-169 | 2787.8 | 0.5 |
| *PA0637* | conserved hypothetical protein | -3566.94 | 9.93E-96 | 4.35E-94 | 422.2 | 0.5 |
| *PA0638* | probable bacteriophage protein | -1080.15 | 3.62E-133 | 2.00E-131 | 705.9 | 1.5 |
| *PA0639* | conserved hypothetical protein | -6114.55 | 3.00E-121 | 1.55E-119 | 693.2 | 0.5 |
| *PA0640* | probable bacteriophage protein | -6022.11 | 1.97E-105 | 9.37E-104 | 687.5 | 0.5 |
| *PA0641* | probable bacteriophage protein | -1678.83 | 3.86E-186 | 3.01E-184 | 3523.1 | 2.3 |
| *PA0643* | hypothetical protein | -9568.88 | 6.09E-124 | 3.19E-122 | 1068.6 | 0.5 |
| *PA0644* | hypothetical protein | -3414.45 | 2.10E-89 | 8.94E-88 | 407.8 | 0.5 |
| *PA0645* | hypothetical protein | -1441.71 | 2.19E-53 | 6.20E-52 | 185.1 | 0.5 |
| *PA0646* | hypothetical protein | -11890.48 | 5.40E-136 | 3.08E-134 | 1307.0 | 0.5 |
| *PA0647* | hypothetical protein | -2565.71 | 1.04E-55 | 2.98E-54 | 312.9 | 0.5 |
| *PA0648* | hypothetical protein | -1613.99 | 1.23E-56 | 3.55E-55 | 207.8 | 0.5 |
| *PA0689* | low-molecular-weight alkaline phosphatase B, LapB | -9.22 | 4.88E-04 | 1.36E-03 | 247.0 | 24.8 |
| *PA0695* | hypothetical protein | 4.28 | 7.81E-05 | 2.50E-04 | 2.1 | 21.8 |
| *PA0701a* |  | -18.43 | 2.45E-17 | 3.61E-16 | 134.8 | 6.7 |
| *PA0711* | hypothetical protein | 4.17 | 5.37E-09 | 3.41E-08 | 13.0 | 59.7 |
| *PA0714* | hypothetical protein | 4.67 | 7.75E-12 | 6.83E-11 | 24.9 | 110.9 |
| *PA0715* | hypothetical protein | -7065.46 | 4.01E-127 | 2.11E-125 | 805.3 | 0.5 |
| *PA0716* | hypothetical protein | -428.61 | 3.81E-109 | 1.84E-107 | 746.2 | 2.0 |
| *PA0718* | hypothetical protein of bacteriophage Pf1 | -204.29 | 3.91E-11 | 3.22E-10 | 27.0 | 0.5 |
| *PA0719* | hypothetical protein of bacteriophage Pf1 | -398.64 | 1.02E-19 | 1.68E-18 | 54.6 | 0.5 |
| *PA0720* | helix destabilizing protein of bacteriophage Pf1 | -196.39 | 1.53E-64 | 4.67E-63 | 258.8 | 1.8 |
| *PA0722* | hypothetical protein of bacteriophage Pf1 | -746.60 | 1.55E-30 | 3.50E-29 | 101.3 | 0.5 |
| *PA0724* | probable coat protein A of bacteriophage Pf1 | -96.05 | 3.48E-25 | 6.99E-24 | 76.3 | 1.5 |
| *PA0725* | hypothetical protein of bacteriophage Pf1 | -6.10 | 1.23E-02 | 2.48E-02 | 2.9 | 1.8 |
| *PA0726* | hypothetical protein of bacteriophage Pf1 | -76.17 | 1.07E-27 | 2.27E-26 | 108.6 | 1.8 |
| *PA0774* | conserved hypothetical protein | 4.40 | 1.15E-22 | 2.18E-21 | 306.4 | 1425.1 |
| *PA0784* | probable transcriptional regulator | -7.61 | 3.37E-76 | 1.23E-74 | 1094.7 | 157.6 |
| *PA0820* | hypothetical protein | -931.83 | 1.89E-97 | 8.60E-96 | 613.4 | 2.5 |
| *PA0845* | CerN | 4.03 | 1.02E-21 | 1.84E-20 | 108.4 | 419.5 |
| *PA0978* | conserved hypothetical protein | 33.32 | 7.92E-83 | 3.05E-81 | 22.8 | 812.1 |
| *PA0979* | conserved hypothetical protein | 26.57 | 9.31E-114 | 4.61E-112 | 28.4 | 744.8 |
| *PA0982* | hypothetical protein | 9.43 | 4.22E-84 | 1.69E-82 | 124.1 | 1173.0 |
| *PA0983* | conserved hypothetical protein | -4.62 | 5.83E-09 | 3.68E-08 | 81.9 | 15.6 |
| *PA0984* | colicin immunity protein | -245.26 | 1.90E-12 | 1.77E-11 | 32.8 | 0.5 |
| *PA0986* | conserved hypothetical protein | 32.25 | 2.81E-48 | 7.60E-47 | 2.2 | 182.3 |
| *PA0987* | conserved hypothetical protein | 13.79 | 1.33E-69 | 4.37E-68 | 29.5 | 391.0 |
| *PA1088* | hypothetical protein | -871.75 | 3.40E-120 | 1.73E-118 | 579.3 | 1.5 |
| *PA1089* | conserved hypothetical protein | -3066.03 | 5.92E-83 | 2.29E-81 | 368.8 | 0.5 |
| *PA1090* | hypothetical protein | -2538.24 | 4.95E-79 | 1.85E-77 | 310.6 | 0.5 |
| *PA1093* | hypothetical protein | -557.33 | 2.34E-169 | 1.62E-167 | 2042.8 | 5.3 |
| *PA1095* | hypothetical protein | -576.32 | 3.56E-165 | 2.33E-163 | 2956.6 | 6.5 |
| *PA1096* | hypothetical protein | -1350.62 | 1.11E-167 | 7.54E-166 | 1542.3 | 3.2 |
| *PA1137* | probable oxidoreductase | 5.97 | 2.58E-27 | 5.42E-26 | 405.8 | 2756.1 |
| *PA1168* | hypothetical protein | 5.51 | 5.64E-26 | 1.14E-24 | 74.8 | 383.2 |
| *PA1169* | probable lipoxygenase | 6.21 | 3.39E-47 | 9.06E-46 | 177.5 | 1106.8 |
| *PA1190* | conserved hypothetical protein | 4.14 | 6.39E-20 | 1.06E-18 | 91.7 | 359.5 |
| *PA1194* | probable amino acid permease | -13.53 | 9.52E-09 | 5.84E-08 | 121.3 | 8.3 |
| *PA1195* | dimethylarginine dimethylaminohydrolase DdaH | -43.33 | 2.68E-30 | 6.03E-29 | 141.1 | 2.7 |
| *PA1196* | transcriptional regulator DdaR | -64.77 | 1.86E-119 | 9.38E-118 | 1163.7 | 20.6 |
| *PA1202* | probable hydrolase | -5.09 | 0.00E+00 | 0.00E+00 | 2303.5 | 551.3 |
| *PA1220* | hypothetical protein | 4.18 | 1.07E-11 | 9.28E-11 | 20.6 | 83.8 |
| *PA1229* | probable transcriptional regulator | -16.93 | 8.51E-14 | 9.14E-13 | 91.5 | 4.8 |
| *PA1231* | conserved hypothetical protein | -6.53 | 7.76E-03 | 1.65E-02 | 3.3 | 1.8 |
| *PA1232* | hypothetical protein | -20.75 | 2.53E-18 | 3.88E-17 | 84.8 | 3.2 |
| *PA1233* | hypothetical protein | -41.74 | 1.63E-17 | 2.41E-16 | 60.6 | 1.8 |
| *PA1239* | hypothetical protein | -14.20 | 1.91E-08 | 1.11E-07 | 29.1 | 2.0 |
| *PA1240* | probable enoyl-CoA hydratase/isomerase | -18.66 | 2.68E-13 | 2.73E-12 | 51.5 | 2.3 |
| *PA1244* | QslA | -11.37 | 6.15E-70 | 2.06E-68 | 630.2 | 55.8 |
| *PA1252* | DpkA | 4.28 | 6.55E-28 | 1.39E-26 | 242.4 | 1083.5 |
| *PA1253* | alpha-ketoglutaric semialdehyde dehydrogenase, LhpG | 6.89 | 1.39E-38 | 3.45E-37 | 44.7 | 286.2 |
| *PA1312* | probable transcriptional regulator | -14.80 | 2.06E-12 | 1.90E-11 | 121.2 | 7.3 |
| *PA1313* | probable major facilitator superfamily (MFS) transporter | -10.66 | 7.73E-06 | 2.99E-05 | 19.8 | 2.0 |
| *PA1314* | hypothetical protein | -18.98 | 1.90E-08 | 1.11E-07 | 26.2 | 1.8 |
| *PA1315* | probable transcriptional regulator | -10.59 | 1.95E-06 | 8.32E-06 | 121.0 | 10.4 |
| *PA1316* | probable major facilitator superfamily (MFS) transporter | -11.85 | 1.47E-16 | 2.03E-15 | 93.1 | 7.5 |
| *PA1346* | hypothetical protein | 4.97 | 3.95E-16 | 5.26E-15 | 23.3 | 107.9 |
| *PA1355* | hypothetical protein | 4.01 | 6.38E-04 | 1.73E-03 | 1.8 | 17.8 |
| *PA1366* | hypothetical protein | -780.17 | 3.57E-83 | 1.39E-81 | 521.1 | 1.5 |
| *PA1368* | hypothetical protein | -1175.21 | 4.84E-45 | 1.27E-43 | 152.7 | 0.5 |
| *PA1369* | hypothetical protein | -577.36 | 2.81E-68 | 9.11E-67 | 395.4 | 1.5 |
| *PA1370* | hypothetical protein | -7321.79 | 5.30E-96 | 2.36E-94 | 826.8 | 0.5 |
| *PA1371* | hypothetical protein | -4458.47 | 1.05E-72 | 3.65E-71 | 515.4 | 0.5 |
| *PA1372* | hypothetical protein | -1168.36 | 6.59E-110 | 3.21E-108 | 2973.0 | 4.7 |
| *PA1395* | hypothetical protein | 4.69 | 9.23E-17 | 1.30E-15 | 35.8 | 148.8 |
| *PA1404* | hypothetical protein | 4.99 | 1.27E-08 | 7.61E-08 | 8.5 | 53.2 |
| *PA1416* | conserved hypothetical protein | 4.08 | 3.06E-24 | 5.99E-23 | 78.6 | 295.6 |
| *PA1428a* |  | -20.57 | 1.68E-12 | 1.58E-11 | 260.8 | 12.9 |
| *PA1471* | hypothetical protein | -659.15 | 6.40E-27 | 1.33E-25 | 89.1 | 0.5 |
| *PA1472* | conserved hypothetical protein | -886.42 | 2.36E-36 | 5.75E-35 | 118.4 | 0.5 |
| *PA1508* | hypothetical protein | -13.47 | 5.10E-15 | 6.07E-14 | 81.3 | 6.3 |
| *PA1540* | conserved hypothetical protein | 4.18 | 1.89E-11 | 1.60E-10 | 23.8 | 91.3 |
| *PA1541* | probable drug efflux transporter | 49.95 | 1.07E-83 | 4.24E-82 | 3.6 | 351.3 |
| *PA1559* | hypothetical protein | 4.69 | 2.36E-29 | 5.23E-28 | 360.0 | 1860.3 |
| *PA1844* | Tse1 | 8.13 | 4.71E-31 | 1.07E-29 | 52.7 | 393.5 |
| *PA1845* | Tsi1 | 5.02 | 3.61E-15 | 4.37E-14 | 48.2 | 219.9 |
| *PA1864* | probable transcriptional regulator | 5.31 | 3.17E-13 | 3.20E-12 | 16.5 | 92.2 |
| *PA1891* | hypothetical protein | 10.39 | 2.08E-68 | 6.77E-67 | 36.8 | 360.9 |
| *PA1892* | hypothetical protein | 5.30 | 4.20E-67 | 1.32E-65 | 199.9 | 1079.3 |
| *PA1893* | hypothetical protein | 5.92 | 4.53E-49 | 1.24E-47 | 511.3 | 3455.4 |
| *PA1894* | hypothetical protein | 6.05 | 1.12E-70 | 3.80E-69 | 543.9 | 3725.4 |
| *PA1895* | hypothetical protein | 9.01 | 5.43E-85 | 2.22E-83 | 403.3 | 4086.2 |
| *PA1896* | hypothetical protein | 10.97 | 5.58E-96 | 2.46E-94 | 367.1 | 4520.1 |
| *PA1897* | hypothetical protein | 8.62 | 1.79E-40 | 4.52E-39 | 414.6 | 4044.8 |
| *PA1913* | hypothetical protein | -4.13 | 2.17E-27 | 4.57E-26 | 653.0 | 170.4 |
| *PA1922* | probable TonB-dependent receptor | 8.43 | 6.61E-07 | 3.01E-06 | 12.1 | 116.7 |
| *PA1931* | probable ferredoxin | -12.11 | 2.46E-06 | 1.03E-05 | 23.0 | 2.1 |
| *PA1932* | probable hydroxylase molybdopterin-containing subunit | -23.82 | 5.89E-17 | 8.48E-16 | 66.4 | 2.5 |
| *PA1933* | probable hydroxylase large subunit | -145.53 | 8.55E-130 | 4.55E-128 | 960.5 | 8.3 |
| *PA1939* | hypothetical protein | -3044.71 | 2.51E-153 | 1.57E-151 | 3314.4 | 3.6 |
| *PA1955* | FapB | 5.48 | 1.97E-04 | 5.86E-04 | 1.3 | 15.2 |
| *PA1977* | hypothetical protein | 5.49 | 2.49E-17 | 3.65E-16 | 24.5 | 123.7 |
| *PA2018* | Resistance-Nodulation-Cell Division (RND) multidrug efflux transporter MexY | 21.57 | 4.32E-84 | 1.72E-82 | 381.7 | 9257.2 |
| *PA2019* | Resistance-Nodulation-Cell Division (RND) multidrug efflux membrane fusion protein MexX precursor | 25.45 | 6.44E-70 | 2.15E-68 | 95.4 | 2438.1 |
| *PA2020* | MexZ | 6.31 | 3.47E-71 | 1.18E-69 | 184.4 | 1198.6 |
| *PA2021* | hypothetical protein | 4.03 | 2.71E-08 | 1.54E-07 | 12.3 | 55.7 |
| *PA2036* | hypothetical protein | 5.21 | 3.92E-11 | 3.22E-10 | 7.7 | 60.3 |
| *PA2037* | hypothetical protein | -8.01 | 1.02E-03 | 2.68E-03 | 157.5 | 17.8 |
| *PA2073* | probable transporter (membrane subunit) | -5.86 | 4.38E-05 | 1.48E-04 | 36.3 | 5.3 |
| *PA2102* | hypothetical protein | -553.08 | 5.11E-23 | 9.75E-22 | 76.3 | 0.5 |
| *PA2103* | probable molybdopterin biosynthesis protein MoeB | -2366.83 | 1.70E-65 | 5.24E-64 | 293.2 | 0.5 |
| *PA2104* | probable cysteine synthase | -1592.76 | 2.50E-51 | 7.02E-50 | 204.3 | 0.5 |
| *PA2105* | probable acetyltransferase | -1511.02 | 2.07E-50 | 5.74E-49 | 195.3 | 0.5 |
| *PA2106* | hypothetical protein | -1197.61 | 4.29E-46 | 1.13E-44 | 156.5 | 0.5 |
| *PA2107* | hypothetical protein | 19.08 | 9.08E-68 | 2.87E-66 | 16.0 | 331.5 |
| *PA2108* | probable decarboxylase | -61.56 | 3.88E-23 | 7.45E-22 | 89.1 | 1.9 |
| *PA2109* | hypothetical protein | -32.33 | 1.04E-31 | 2.40E-30 | 146.8 | 5.4 |
| *PA2119* | alcohol dehydrogenase (Zn-dependent) | -83.67 | 1.08E-07 | 5.65E-07 | 4443.7 | 62.1 |
| *PA2134* | hypothetical protein | 6.99 | 9.31E-08 | 4.90E-07 | 1.8 | 30.3 |
| *PA2135* | probable transporter | 4.43 | 1.02E-05 | 3.86E-05 | 5.5 | 44.3 |
| *PA2136* | hypothetical protein | -9.72 | 2.19E-13 | 2.25E-12 | 88.5 | 8.5 |
| *PA2141* | hypothetical protein | 7.15 | 1.49E-05 | 5.48E-05 | 1.5 | 22.1 |
| *PA2143* | hypothetical protein | 4.52 | 1.72E-06 | 7.40E-06 | 3.5 | 33.4 |
| *PA2146* | conserved hypothetical protein | 5.34 | 1.77E-03 | 4.40E-03 | 1.3 | 13.2 |
| *PA2150* | conserved hypothetical protein | 4.72 | 2.87E-09 | 1.89E-08 | 9.6 | 58.1 |
| *PA2154* | conserved hypothetical protein | 8.52 | 4.93E-09 | 3.15E-08 | 1.8 | 32.3 |
| *PA2155* | probable phospholipase | 4.21 | 7.00E-08 | 3.77E-07 | 7.1 | 44.1 |
| *PA2156* | conserved hypothetical protein | 4.94 | 2.12E-07 | 1.06E-06 | 3.0 | 34.6 |
| *PA2158* | probable alcohol dehydrogenase (Zn-dependent) | -7.24 | 2.87E-05 | 1.00E-04 | 23.7 | 2.9 |
| *PA2160* | probable glycosyl hydrolase | 5.20 | 2.08E-21 | 3.73E-20 | 35.0 | 164.8 |
| *PA2161* | hypothetical protein | 4.67 | 1.90E-02 | 3.65E-02 | 1.0 | 9.3 |
| *PA2163* | hypothetical protein | 6.46 | 3.12E-20 | 5.31E-19 | 19.7 | 124.7 |
| *PA2168* | hypothetical protein | 5.63 | 7.39E-08 | 3.96E-07 | 3.3 | 38.7 |
| *PA2169* | hypothetical protein | 12.14 | 2.90E-21 | 5.17E-20 | 3.0 | 86.5 |
| *PA2171* | hypothetical protein | 14.30 | 7.69E-29 | 1.67E-27 | 9.5 | 168.5 |
| *PA2172* | hypothetical protein | 5.25 | 5.61E-22 | 1.02E-20 | 39.8 | 186.8 |
| *PA2175* | hypothetical protein | 9.84 | 3.34E-16 | 4.45E-15 | 15.6 | 152.6 |
| *PA2176* | hypothetical protein | 7.85 | 4.93E-52 | 1.39E-50 | 53.8 | 392.8 |
| *PA2182* | hypothetical protein | -100.24 | 7.53E-07 | 3.40E-06 | 8.1 | 0.7 |
| *PA2183* | hypothetical protein | -28.43 | 3.75E-08 | 2.10E-07 | 21.0 | 1.7 |
| *PA2184* | conserved hypothetical protein | -9.68 | 5.49E-05 | 1.81E-04 | 17.1 | 2.3 |
| *PA2188* | probable alcohol dehydrogenase (Zn-dependent) | -131.80 | 2.57E-07 | 1.26E-06 | 15.3 | 0.7 |
| *PA2190* | conserved hypothetical protein | -18.54 | 2.89E-08 | 1.64E-07 | 25.6 | 1.9 |
| *PA2192* | conserved hypothetical protein | -91.44 | 2.86E-06 | 1.19E-05 | 8.3 | 0.7 |
| *PA2205* | hypothetical protein | 5.42 | 1.98E-18 | 3.07E-17 | 41.8 | 206.3 |
| *PA2221* | conserved hypothetical protein | 5.60 | 1.04E-24 | 2.04E-23 | 38.5 | 194.0 |
| *PA2260* | hypothetical protein | 6.78 | 3.65E-66 | 1.14E-64 | 84.7 | 544.3 |
| *PA2261* | probable 2-ketogluconate kinase | 7.37 | 3.91E-73 | 1.37E-71 | 84.8 | 595.8 |
| *PA2262* | probable 2-ketogluconate transporter | 4.21 | 4.26E-21 | 7.48E-20 | 81.7 | 316.8 |
| *PA2263* | probable 2-hydroxyacid dehydrogenase | 6.60 | 2.01E-26 | 4.11E-25 | 46.2 | 279.9 |
| *PA2294* | probable ATP-binding component of ABC transporter | -6.57 | 6.02E-07 | 2.76E-06 | 35.0 | 6.0 |
| *PA2295* | probable permease of ABC transporter | -6.95 | 5.13E-03 | 1.15E-02 | 4.1 | 1.9 |
| *PA2296* | hypothetical protein | -7.70 | 1.27E-06 | 5.55E-06 | 48.3 | 5.8 |
| *PA2319* | probable transposase | 6.67 | 2.88E-03 | 6.83E-03 | 0.9 | 8.2 |
| *PA2324* | hypothetical protein | 6.33 | 1.20E-22 | 2.27E-21 | 30.3 | 175.2 |
| *PA2333* | probable sulfatase | -9.66 | 2.04E-09 | 1.37E-08 | 53.0 | 6.2 |
| *PA2334* | probable transcriptional regulator | -6.04 | 1.09E-03 | 2.83E-03 | 12.6 | 2.8 |
| *PA2335* | probable TonB-dependent receptor | -18.93 | 3.30E-08 | 1.86E-07 | 26.2 | 2.1 |
| *PA2336* | hypothetical protein | -5.40 | 3.46E-03 | 7.98E-03 | 5.2 | 4.0 |
| *PA2347* | hypothetical protein | 4.22 | 2.65E-07 | 1.30E-06 | 8.2 | 44.3 |
| *PA2355* | probable FMNH2-dependent monooxygenase | 4.84 | 7.85E-10 | 5.51E-09 | 17.3 | 85.1 |
| *PA2414* | L-sorbosone dehydrogenase | 5.63 | 1.41E-70 | 4.76E-69 | 130.8 | 726.3 |
| *PA2415* | hypothetical protein | 7.83 | 2.29E-18 | 3.52E-17 | 10.4 | 97.8 |
| *PA2420* | probable porin | 4.40 | 1.69E-12 | 1.59E-11 | 25.6 | 99.9 |
| *PA2421* | hypothetical protein | 31.29 | 1.08E-69 | 3.58E-68 | 3.8 | 267.6 |
| *PA2422* | hypothetical protein | 21.16 | 3.11E-38 | 7.70E-37 | 2.4 | 117.5 |
| *PA2427* | hypothetical protein | -4.25 | 4.34E-03 | 9.83E-03 | 13.9 | 4.9 |
| *PA2433* | hypothetical protein | 4.34 | 1.31E-14 | 1.50E-13 | 42.8 | 166.8 |
| *PA2458* | hypothetical protein | -5.26 | 7.97E-31 | 1.81E-29 | 395.9 | 75.4 |
| *PA2459* | hypothetical protein | -4.86 | 4.96E-04 | 1.38E-03 | 56.3 | 11.9 |
| *PA2460* | hypothetical protein | -1057.01 | 6.46E-30 | 1.44E-28 | 139.4 | 0.8 |
| *PA2461* | hypothetical protein | -1159.51 | 9.47E-38 | 2.34E-36 | 150.9 | 0.8 |
| *PA2462* | hypothetical protein | -4.82 | 1.26E-12 | 1.21E-11 | 6509.8 | 1784.5 |
| *PA2465* | hypothetical protein | 4.31 | 5.41E-07 | 2.51E-06 | 6.3 | 40.4 |
| *PA2472* | probable major facilitator superfamily (MFS) transporter | 4.08 | 1.71E-05 | 6.24E-05 | 7.1 | 38.6 |
| *PA2473* | maleylpyruvate isomerase | 6.45 | 9.84E-07 | 4.38E-06 | 1.7 | 25.3 |
| *PA2485* | hypothetical protein | 4.62 | 3.40E-15 | 4.14E-14 | 28.2 | 116.7 |
| *PA2487* | hypothetical protein | 4.12 | 8.20E-06 | 3.15E-05 | 5.8 | 37.7 |
| *PA2496* | hypothetical protein | 6.62 | 1.81E-44 | 4.69E-43 | 83.1 | 512.9 |
| *PA2497* | probable transcriptional regulator | 8.94 | 1.36E-84 | 5.53E-83 | 91.4 | 789.2 |
| *PA2498* | conserved hypothetical protein | 4.18 | 8.53E-11 | 6.68E-10 | 25.3 | 95.8 |
| *PA2499* | probable deaminase | 5.35 | 1.34E-13 | 1.40E-12 | 12.0 | 70.9 |
| *PA2506* | hypothetical protein | 6.31 | 4.44E-03 | 1.00E-02 | 0.9 | 9.0 |
| *PA2562* | hypothetical protein | 4.90 | 1.10E-31 | 2.52E-30 | 578.8 | 3260.4 |
| *PA2564* | hypothetical protein | -16.48 | 6.85E-15 | 8.01E-14 | 122.6 | 7.0 |
| *PA2565* | hypothetical protein | -218.52 | 3.27E-10 | 2.38E-09 | 28.6 | 0.8 |
| *PA2566* | conserved hypothetical protein | -34.44 | 3.42E-26 | 6.94E-25 | 116.2 | 3.1 |
| *PA2569* | hypothetical protein | 6.57 | 3.17E-19 | 5.12E-18 | 29.3 | 175.1 |
| *PA2594* | conserved hypothetical protein | -10.80 | 5.56E-05 | 1.84E-04 | 228.0 | 20.2 |
| *PA2595* | conserved hypothetical protein | -9.63 | 2.25E-08 | 1.30E-07 | 75.4 | 7.2 |
| *PA2596* | conserved hypothetical protein | -18.53 | 3.88E-10 | 2.81E-09 | 38.8 | 2.5 |
| *PA2597* | hypothetical protein | -24.23 | 1.09E-16 | 1.52E-15 | 67.8 | 3.0 |
| *PA2598* | hypothetical protein | -75.33 | 2.67E-29 | 5.89E-28 | 107.9 | 2.3 |
| *PA2599* | conserved hypothetical protein | -17.38 | 2.94E-13 | 2.97E-12 | 84.3 | 4.5 |
| *PA2600* | hypothetical protein | -21.70 | 4.93E-16 | 6.53E-15 | 89.5 | 3.8 |
| *PA2601* | probable transcriptional regulator | -18.99 | 5.55E-12 | 4.97E-11 | 216.3 | 11.8 |
| *PA2602* | 3-mercaptopropionate dioxygenase | -33.18 | 1.41E-09 | 9.59E-09 | 25.4 | 2.1 |
| *PA2603* | probable thiosulfate sulfurtransferase | -32.51 | 4.89E-34 | 1.18E-32 | 226.1 | 7.4 |
| *PA2667* | MvaU | 4.34 | 3.26E-29 | 7.17E-28 | 852.6 | 4336.4 |
| *PA2702* | Tse2 | 4.73 | 1.14E-28 | 2.46E-27 | 89.6 | 399.3 |
| *PA2703* | Tsi2 | 4.72 | 2.85E-19 | 4.61E-18 | 39.5 | 167.8 |
| *PA2730* | hypothetical protein | -705.88 | 1.95E-135 | 1.10E-133 | 847.8 | 3.8 |
| *PA2732* | hypothetical protein | -984.41 | 9.43E-176 | 7.07E-174 | 2631.8 | 5.0 |
| *PA2733* | conserved hypothetical protein | -3493.84 | 3.56E-84 | 1.44E-82 | 416.0 | 1.0 |
| *PA2734* | hypothetical protein | -1106.59 | 1.51E-172 | 1.09E-170 | 1830.8 | 4.3 |
| *PA2735* | type I HsdM methyltransferase | -4619.26 | 4.87E-182 | 3.75E-180 | 2856.1 | 2.8 |
| *PA2736* | hypothetical protein | -1611.53 | 4.86E-57 | 1.41E-55 | 205.4 | 1.0 |
| *PA2764* | hypothetical protein | -10.95 | 4.76E-05 | 1.59E-04 | 216.6 | 19.1 |
| *PA2771* | diguanylate cyclase with a self-blocked I-site, Dcsbis | -17.32 | 4.39E-07 | 2.07E-06 | 348.4 | 20.4 |
| *PA2772* | hypothetical protein | -26.79 | 3.68E-21 | 6.54E-20 | 91.4 | 3.3 |
| *PA2772a* |  | -14.29 | 5.81E-04 | 1.60E-03 | 5.5 | 2.2 |
| *PA2774* | Tse4 | -4.29 | 2.57E-04 | 7.53E-04 | 212.1 | 47.9 |
| *PA2775* | Tsi4 | -4.71 | 1.25E-02 | 2.52E-02 | 82.7 | 16.3 |
| *PA2777* | conserved hypothetical protein | 4.20 | 2.94E-26 | 6.01E-25 | 108.6 | 441.3 |
| *PA2792* | hypothetical protein | 4.28 | 4.21E-28 | 9.02E-27 | 145.3 | 618.5 |
| *PA2793* | hypothetical protein | 4.03 | 3.77E-24 | 7.35E-23 | 270.3 | 1140.6 |
| *PA3054* | hypothetical protein | -4.35 | 2.40E-32 | 5.64E-31 | 1032.9 | 263.1 |
| *PA3065* | hypothetical protein | -235.87 | 1.43E-12 | 1.35E-11 | 31.9 | 1.3 |
| *PA3066* | hypothetical protein | -138.59 | 3.53E-29 | 7.74E-28 | 107.8 | 2.8 |
| *PA3067* | probable transcriptional regulator | -447.80 | 4.91E-22 | 8.99E-21 | 62.0 | 1.3 |
| *PA3142* | integrase | -73.11 | 1.82E-48 | 4.94E-47 | 227.4 | 5.0 |
| *PA3143* | transposase | -40.72 | 8.26E-33 | 1.96E-31 | 178.4 | 5.7 |
| *PA3157* | probable acetyltransferase | -8313.07 | 1.75E-145 | 1.05E-143 | 947.3 | 1.3 |
| *PA3237* | hypothetical protein | 4.73 | 1.36E-03 | 3.47E-03 | 1.3 | 14.3 |
| *PA3362* | hypothetical protein | -32.07 | 8.15E-34 | 1.95E-32 | 241.2 | 8.7 |
| *PA3434* | probable transposase | -114.27 | 5.12E-07 | 2.39E-06 | 12.4 | 1.5 |
| *PA3486* | VgrG4b | -4.52 | 5.23E-27 | 1.09E-25 | 813.8 | 195.0 |
| *PA3488* | Tli5 | -3889.09 | 2.18E-73 | 7.66E-72 | 455.0 | 1.6 |
| *PA3499* | hypothetical protein | 4.48 | 2.06E-07 | 1.03E-06 | 7.4 | 43.0 |
| *PA3661* | hypothetical protein | 20.72 | 1.92E-54 | 5.48E-53 | 6.6 | 223.1 |
| *PA3733a* |  | 12.41 | 8.52E-82 | 3.26E-80 | 55.9 | 653.2 |
| *PA3819* | conserved hypothetical protein | 5.61 | 2.18E-71 | 7.49E-70 | 1554.5 | 11326.3 |
| *PA3865a* |  | -133.54 | 4.29E-30 | 9.62E-29 | 103.0 | 2.6 |
| *PA3866* | Pyocin S4 | -2108.36 | 1.99E-173 | 1.45E-171 | 3337.5 | 4.2 |
| *PA3867* | probable DNA invertase | -543.39 | 6.48E-25 | 1.29E-23 | 75.4 | 1.7 |
| *PA3868* | hypothetical protein | -285.50 | 6.21E-15 | 7.30E-14 | 39.0 | 1.7 |
| *PA3869* | hypothetical protein | -190.77 | 1.87E-10 | 1.40E-09 | 25.5 | 1.7 |
| *PA3928* | hypothetical protein | -4.09 | 6.12E-06 | 2.41E-05 | 45.3 | 9.3 |
| *PA3939* | hypothetical protein | -22.11 | 5.98E-27 | 1.24E-25 | 129.3 | 7.1 |
| *PA3961* | probable ATP-dependent helicase | -5.08 | 6.29E-20 | 1.05E-18 | 996.9 | 217.2 |
| *PA3993* | probable transposase | -5.31 | 1.22E-02 | 2.47E-02 | 3.3 | 3.5 |
| *PA4095* | hypothetical protein | 4.81 | 4.07E-07 | 1.93E-06 | 3.3 | 35.6 |
| *PA4128* | conserved hypothetical protein | -5.13 | 6.40E-68 | 2.06E-66 | 757.9 | 157.6 |
| *PA4129* | hypothetical protein | -26.22 | 7.87E-112 | 3.87E-110 | 2317.8 | 98.0 |
| *PA4130* | probable sulfite or nitrite reductase | -27.59 | 1.89E-132 | 1.04E-130 | 8956.6 | 421.8 |
| *PA4131* | probable iron-sulfur protein | -80.41 | 2.63E-178 | 2.00E-176 | 12446.0 | 188.8 |
| *PA4132* | conserved hypothetical protein | -25.52 | 1.87E-131 | 1.01E-129 | 9167.1 | 469.8 |
| *PA4133* | cytochrome c oxidase subunit (cbb3-type) | -72.01 | 6.65E-108 | 3.18E-106 | 7541.6 | 129.2 |
| *PA4134* | hypothetical protein | -24.62 | 1.93E-83 | 7.60E-82 | 545.8 | 21.8 |
| *PA4139* | hypothetical protein | 13.45 | 2.99E-41 | 7.57E-40 | 128.8 | 1757.3 |
| *PA4140* | hypothetical protein | 8.61 | 6.73E-43 | 1.73E-41 | 87.1 | 713.4 |
| *PA4141* | hypothetical protein | 4.31 | 1.28E-15 | 1.60E-14 | 1213.9 | 6398.8 |
| *PA4142* | probable secretion protein | 7.67 | 2.61E-64 | 7.90E-63 | 116.2 | 877.4 |
| *PA4143* | probable toxin transporter | 6.50 | 1.39E-74 | 4.97E-73 | 136.8 | 874.1 |
| *PA4144* | probable outer membrane protein precursor | 6.91 | 2.29E-58 | 6.73E-57 | 68.3 | 435.1 |
| *PA4148* | probable short-chain dehydrogenase | -9.67 | 9.04E-07 | 4.05E-06 | 26.1 | 3.5 |
| *PA4149* | conserved hypothetical protein | -21.65 | 2.49E-16 | 3.33E-15 | 74.7 | 3.8 |
| *PA4150* | probable dehydrogenase E1 component | -10.75 | 9.89E-10 | 6.90E-09 | 68.1 | 6.9 |
| *PA4152* | probable hydrolase | -198.48 | 1.43E-08 | 8.52E-08 | 25.4 | 1.8 |
| *PA4153* | 2,3-butanediol dehydrogenase | -14.17 | 7.63E-10 | 5.37E-09 | 39.2 | 3.7 |
| *PA4173* | conserved hypothetical protein | 8.87 | 1.77E-13 | 1.84E-12 | 3.0 | 51.7 |
| *PA4191* | isopenicillin-N synthase | -17.91 | 1.00E-11 | 8.74E-11 | 206.9 | 12.6 |
| *PA4192* | probable ATP-binding component of ABC transporter | -20.82 | 8.30E-14 | 8.93E-13 | 58.3 | 3.7 |
| *PA4193* | probable permease of ABC transporter | -8.98 | 2.68E-07 | 1.31E-06 | 43.4 | 5.3 |
| *PA4194* | probable permease of ABC transporter | -15.57 | 3.18E-09 | 2.08E-08 | 43.2 | 3.7 |
| *PA4195* | probable binding protein component of ABC transporter | -12.73 | 5.44E-12 | 4.88E-11 | 88.4 | 7.6 |
| *PA4200* | hypothetical protein | -6.11 | 2.45E-72 | 8.46E-71 | 1453.0 | 273.7 |
| *PA4220* | hypothetical protein | -6.48 | 4.71E-10 | 3.37E-09 | 82.5 | 12.2 |
| *PA4222* | probable ATP-binding component of ABC transporter | -13.18 | 5.94E-14 | 6.50E-13 | 827.1 | 64.2 |
| *PA4223* | probable ATP-binding component of ABC transporter | -14.84 | 2.03E-10 | 1.52E-09 | 833.6 | 57.5 |
| *PA4289* | probable transporter | 4.92 | 6.46E-39 | 1.61E-37 | 174.8 | 865.4 |
| *PA4429* | probable cytochrome c1 precursor | -4.13 | 0.00E+00 | 0.00E+00 | 12387.1 | 3952.7 |
| *PA4430* | probable cytochrome b | -4.49 | 0.00E+00 | 0.00E+00 | 13876.3 | 4159.5 |
| *PA4431* | probable iron-sulfur protein | -4.08 | 0.00E+00 | 0.00E+00 | 7199.2 | 2406.1 |
| *PA4514* | probable outer membrane receptor for iron transport | -11.83 | 9.13E-76 | 3.29E-74 | 555.1 | 46.2 |
| *PA4582* | conserved hypothetical protein | 8.69 | 7.67E-68 | 2.46E-66 | 109.8 | 924.3 |
| *PA4583* | conserved hypothetical protein | 5.26 | 3.57E-32 | 8.33E-31 | 189.5 | 1002.9 |
| *PA4584* | conserved hypothetical protein | 4.40 | 1.02E-26 | 2.10E-25 | 128.2 | 545.9 |
| *PA4624* | cyclic diguanylate-regulated TPS partner B, CdrB | 5.85 | 6.89E-37 | 1.69E-35 | 377.4 | 2473.6 |
| *PA4625* | cyclic diguanylate-regulated TPS partner A, CdrA | 4.41 | 3.01E-18 | 4.60E-17 | 971.8 | 5208.9 |
| *PA4802* | hypothetical protein | -10.24 | 1.53E-07 | 7.83E-07 | 94.9 | 10.0 |
| *PA4918* | nicotinamidase, PcnA | -26.60 | 5.78E-131 | 3.11E-129 | 2739.5 | 114.1 |
| *PA4933* | hypothetical protein | -5.10 | 0.00E+00 | 0.00E+00 | 20705.3 | 6419.7 |
| *PA4985* | Uncharacterized protein | 4.80 | 1.41E-21 | 2.55E-20 | 52.1 | 221.4 |
| *PA5088* | type VI secretion lipase immunity protein, Tli5b3 | -5.41 | 1.07E-02 | 2.19E-02 | 210.8 | 37.7 |
| *PA5212* | hypothetical protein | 4.59 | 3.61E-29 | 7.87E-28 | 291.6 | 1437.3 |
| *PA5264* | hypothetical protein | -14.57 | 3.57E-05 | 1.22E-04 | 506.3 | 36.8 |
| *PA5265* | hypothetical protein | -25.02 | 1.86E-05 | 6.71E-05 | 1251.0 | 56.4 |
| *PA5318* | hypothetical protein | -4.71 | 1.13E-12 | 1.09E-11 | 175.4 | 35.1 |
| *pchA* | salicylate biosynthesis isochorismate synthase | -4.63 | 2.91E-11 | 2.43E-10 | 532.6 | 118.8 |
| *pchB* | salicylate biosynthesis protein PchB | -6.50 | 4.31E-07 | 2.03E-06 | 90.3 | 12.7 |
| *pchC* | pyochelin biosynthetic protein PchC | -5.43 | 2.62E-07 | 1.28E-06 | 118.3 | 20.4 |
| *pchD* | pyochelin biosynthesis protein PchD | -5.94 | 5.42E-11 | 4.34E-10 | 308.3 | 49.1 |
| *pchE* | dihydroaeruginoic acid synthetase | -19.72 | 7.74E-16 | 9.93E-15 | 4027.4 | 243.3 |
| *pchF* | pyochelin synthetase | -21.72 | 9.75E-16 | 1.24E-14 | 5378.6 | 296.2 |
| *pchG* | pyochelin biosynthetic protein PchG | -20.82 | 1.17E-12 | 1.12E-11 | 841.3 | 41.4 |
| *pelD* | PelD | 5.55 | 2.27E-36 | 5.54E-35 | 104.8 | 561.9 |
| *pelE* | PelE | 4.80 | 3.74E-21 | 6.62E-20 | 65.3 | 280.4 |
| *pelF* | PelF | 5.00 | 3.82E-28 | 8.20E-27 | 88.6 | 414.8 |
| *phzC2* | phenazine biosynthesis protein PhzC | -5.10 | 9.25E-06 | 3.53E-05 | 48.3 | 9.3 |
| *phzG2* | probable pyridoxamine 5'-phosphate oxidase | 6.38 | 7.77E-19 | 1.23E-17 | 17.9 | 111.5 |
| *pilA* | type 4 fimbrial precursor PilA | -961.48 | 1.98E-223 | 1.61E-221 | 29476.2 | 40.7 |
| *piv* | protease IV | -4.71 | 0.00E+00 | 0.00E+00 | 4351.8 | 1129.2 |
| *pldA* |  | -665.68 | 1.04E-140 | 6.08E-139 | 2396.5 | 6.0 |
| *pncB1* | nicotinate phosphoribosyltransferase | -11.15 | 2.78E-96 | 1.26E-94 | 4138.0 | 457.0 |
| *pqqB* | pyrroloquinoline quinone biosynthesis protein B | 4.43 | 1.29E-26 | 2.65E-25 | 130.5 | 566.2 |
| *pqqC* | pyrroloquinoline quinone biosynthesis protein C | 4.61 | 1.25E-29 | 2.78E-28 | 114.6 | 503.7 |
| *pqqD* | pyrroloquinoline quinone biosynthesis protein D | 8.25 | 1.59E-13 | 1.65E-12 | 11.3 | 109.3 |
| *pqqH* | PqqH | 5.48 | 8.37E-42 | 2.13E-40 | 164.6 | 909.7 |
| *prrF1* | regulatory RNA PrrF1 | -6.48 | 2.73E-02 | 4.99E-02 | 1.9 | 3.6 |
| *purA* | adenylosuccinate synthetase | -4.11 | 0.00E+00 | 0.00E+00 | 7273.8 | 2373.6 |
| *putA* | proline dehydrogenase PutA | -15.94 | 0.00E+00 | 0.00E+00 | 16030.1 | 1475.8 |
| *putP* | sodium/proline symporter PutP | -6.61 | 0.00E+00 | 0.00E+00 | 6975.0 | 1377.6 |
| *pvcA* | paerucumarin biosynthesis protein PvcA | 5.62 | 1.18E-08 | 7.12E-08 | 3.6 | 38.9 |
| *pvcD* | paerucumarin biosynthesis protein PvcD | 5.27 | 2.56E-06 | 1.08E-05 | 2.7 | 31.7 |
| *pvdD* | pyoverdine synthetase D | -10.37 | 9.29E-66 | 2.88E-64 | 493.9 | 48.6 |
| *pvdE* | pyoverdine biosynthesis protein PvdE | -6.12 | 7.35E-13 | 7.19E-12 | 104.6 | 16.4 |
| *pvdJ* | PvdJ | -8.68 | 1.09E-44 | 2.84E-43 | 495.5 | 59.3 |
| *pyoS5* | pyocin S5 | -13501.20 | 1.79E-120 | 9.20E-119 | 1480.2 | 3.1 |
| *pys2* | pyocin S2 | -42.07 | 2.15E-07 | 1.07E-06 | 1776.8 | 48.9 |
| *qscR* | quorum-sensing control repressor | 12.05 | 1.63E-89 | 6.98E-88 | 95.4 | 1111.8 |
| *rgsA* | RgsA | -5.11 | 3.87E-24 | 7.53E-23 | 420.6 | 82.7 |
| *rplD* | 50S ribosomal protein L4 | -4.16 | 0.00E+00 | 0.00E+00 | 22130.3 | 8228.0 |
| *rplI* | 50S ribosomal protein L9 | -4.74 | 0.00E+00 | 0.00E+00 | 16308.1 | 5201.2 |
| *rplW* | 50S ribosomal protein L23 | -4.07 | 0.00E+00 | 0.00E+00 | 6590.8 | 2173.5 |
| *rpmJ* | 50S ribosomal protein L36 | -4.30 | 8.32E-04 | 2.21E-03 | 25.9 | 6.9 |
| *rpsB* | 30S ribosomal protein S2 | -4.01 | 0.00E+00 | 0.00E+00 | 23915.1 | 9418.9 |
| *rpsF* | 30S ribosomal protein S6 | -6.77 | 0.00E+00 | 0.00E+00 | 15811.9 | 3473.7 |
| *rpsG* | 30S ribosomal protein S7 | -4.20 | 0.00E+00 | 0.00E+00 | 15490.9 | 5519.3 |
| *rpsR* | 30S ribosomal protein S18 | -6.70 | 0.00E+00 | 0.00E+00 | 5780.8 | 1076.2 |
| *sahH* | S-adenosyl-L-homocysteine hydrolase | -4.89 | 0.00E+00 | 0.00E+00 | 10424.3 | 2872.0 |
| *secB* | secretion protein SecB | -5.62 | 0.00E+00 | 0.00E+00 | 4075.4 | 904.6 |
| *speC* | ornithine decarboxylase | -5.47 | 0.00E+00 | 0.00E+00 | 2615.9 | 579.9 |
| *speD* | S-adenosylmethionine decarboxylase proenzyme | -5.77 | 1.05E-13 | 1.11E-12 | 1718.5 | 350.5 |
| *treA* | periplasmic trehalase precursor | -8.71 | 1.37E-08 | 8.19E-08 | 54.0 | 7.3 |
| *tsf* | elongation factor Ts | -4.83 | 0.00E+00 | 0.00E+00 | 17515.2 | 5507.1 |
| *tufA* | elongation factor Tu | -7.13 | 0.00E+00 | 0.00E+00 | 45756.2 | 10041.1 |
| *ureA* | urease gamma subunit | -4.08 | 1.88E-12 | 1.76E-11 | 109.9 | 24.0 |
| *vgrG1* | VgrG1 | 7.12 | 6.55E-50 | 1.81E-48 | 732.8 | 6297.0 |
| *waaL* | O-antigen ligase, WaaL | -8.00 | 4.89E-11 | 3.94E-10 | 545.0 | 71.8 |
| *wbpA* | UDP-N-acetyl-d-glucosamine 6-Dehydrogenase | -1275.14 | 1.38E-201 | 1.09E-199 | 6270.7 | 7.2 |
| *wbpB* | UDP-2-acetamido-2-deoxy-d-glucuronic acid 3-dehydrogenase, WbpB | -1832.88 | 4.04E-166 | 2.71E-164 | 3815.0 | 4.8 |
| *wbpD* | UDP-2-acetamido-3-amino-2,3-dideoxy-d-glucuronic acid N-acetyltransferase, WbpD | -2685.85 | 1.01E-164 | 6.53E-163 | 1683.3 | 3.4 |
| *wbpE* | UDP-2-acetamido-2-dideoxy-d-ribo-hex-3-uluronic acid transaminase, wbpE | -2156.03 | 8.55E-175 | 6.33E-173 | 4611.0 | 4.8 |
| *wbpG* | LPS biosynthesis protein WbpG | -2523.51 | 6.05E-143 | 3.59E-141 | 4083.4 | 4.2 |
| *wbpH* | probable glycosyltransferase WbpH | -1978.78 | 6.88E-168 | 4.72E-166 | 2238.8 | 3.7 |
| *wbpI* | UDP-N-acetylglucosamine 2-epimerase WbpI | -2058.27 | 1.13E-148 | 6.90E-147 | 3268.5 | 4.3 |
| *wbpJ* | probable glycosyl transferase WbpJ | -1336.39 | 1.54E-163 | 9.86E-162 | 1523.7 | 4.3 |
| *wbpK* | probable NAD-dependent epimerase/dehydratase WbpK | -1800.55 | 3.27E-151 | 2.03E-149 | 1150.5 | 3.7 |
| *wbpL* | glycosyltransferase WbpL | -1912.92 | 5.30E-166 | 3.51E-164 | 1218.6 | 3.8 |
| *wzx* | O-antigen translocase | -638.58 | 6.50E-100 | 3.01E-98 | 435.9 | 3.4 |
| *wzy* | B-band O-antigen polymerase | -6615.39 | 4.37E-140 | 2.54E-138 | 757.6 | 3.2 |
| *wzz* | O-antigen chain length regulator | -655.23 | 1.18E-153 | 7.50E-152 | 1113.7 | 4.5 |
| *lasB* | elastase LasB | 2.60 | 1.62E-06 | 7.02E-06 | 4484.2 | 15730.4 |
| *rhlA* | rhamnosyltransferase chain A | 2.74 | 1.12E-15 | 1.41E-14 | 592.2 | 1887.9 |
| *rhlB* | rhamnosyltransferase chain B | 3.05 | 3.80E-17 | 5.51E-16 | 294.8 | 940.1 |

**Table S4.** Full list of genes significantly regulated in LYSZa7 comparing to *P. aeruginosa* PAO1 (fold change ≧ 2, FDR adjusted p-value < 0.05); means: normalized mean count of the sample group.
